# Integrating Biomarkers into Cervical Cancer Screening—Advances in Diagnosis and Risk Prediction: A Narrative Review

**DOI:** 10.3390/diagnostics15243231

**Published:** 2025-12-17

**Authors:** Tudor Gisca, Daniela Roxana Matasariu, Alexandra Ursache, Demetra Gabriela Socolov, Ioana-Sadiye Scripcariu, Alina Fudulu, Ecaterina Tomaziu-Todosia Anton, Anca Botezatu

**Affiliations:** 1 Department of Obstetrics and Gynecology, Grigore T. Popa University of Medicine and Pharmacy, 700115 Iasi, Romania; tudor-catalin.gisca@umfiasi.ro (T.G.); alexandra.ursache@umfiasi.ro (A.U.); demetra.socolov@umfiasi.ro (D.G.S.); ioana.scripcariu@umfiasi.ro (I.-S.S.); tomaziu-todosia.ecaterina@d.umfiasi.ro (E.T.-T.A.); 2 Department of Obstetrics and Gynecology, Cuza Voda Hospital, 700038 Iasi, Romania; 3 Stefan S Nicolau Institute of Virology, Romanian Academy, 030304 Bucharest, Romania; alina.fudulu@virology.ro (A.F.); anca.botezatu@virology.ro (A.B.)

**Keywords:** cervical cancer, human papillomavirus (HPV), prognostic biomarkers, p16 immunohistochemistry, cervical carcinogenesis, HPV-related dysplasia, cervical cancer screening, DNA methylation, microbiota

## Abstract

**Background:** Cervical cancer remains a major global health challenge, ranking fourth among malignancies in women, with an estimated 660,000 new cases and 350,000 deaths in 2022. Despite advances in vaccination and screening, incidence and mortality remain disproportionately high in low- and middle-income countries. The disease is strongly linked to persistent infection with high-risk human papillomavirus (HPV) types, predominantly HPV 16 and 18, whose E6 and E7 oncoproteins drive cervical intraepithelial neoplasia (CIN) and invasive cancer. This review summarizes current evidence on clinically relevant biomarkers in HPV-associated CIN and cervical cancer, emphasizing their role in screening, risk stratification, and disease management. **Methods:** We analyzed the recent literature focusing on validated and emerging biomarkers with potential clinical applications in HPV-related cervical disease. **Results:** Biomarkers are essential tools for improving early detection, assessment of progression risk, and personalized management. Established markers such as p16 immunostaining, p16/Ki-67 dual staining, and HPV E6/E7 mRNA assays increase diagnostic accuracy and reduce overtreatment. Prognostic indicators, including squamous cell carcinoma antigen (SCC-Ag) and telomerase activity, provide information on tumor burden and recurrence risk. Novel approaches—such as DNA methylation panels, HPV viral load quantification, ncRNAs, and cervico-vaginal microbiota profiling—show promise in refining risk assessment and supporting non-invasive follow-up strategies. **Conclusions:** The integration of validated biomarkers into clinical practice facilitates more effective triage, individualized treatment decisions, and optimal use of healthcare resources. Emerging biomarkers, once validated, could further improve precision in predicting lesion outcomes, ultimately reducing the global burden of cervical cancer and improving survival.

## 1. Introduction

Cervical cancer ranks fourth among neoplasia affecting women, accounting for approximately 8% of total cancer cases and deaths. The incidence in low- and middle-income countries is disproportionately high, with rates significantly higher than in high-income countries [1]. According to data from GLOBOCAN on cervical cancer, there were approximately 660,000 new cases and 350,000 deaths in 2022, with over 19.3 per 100,000 of these cases occurring in low- and middle-income countries. Most of the cases are found in Africa, Southeast Asia, and Eastern Europe. In Romania, the incidence rate is approximately three times the European Union average, being one of the highest in this region. In Romania, it is the third most frequently diagnosed type of cancer, with a prevalence of 11,278 (115.3 per 100,000). A total of 3368 new cases of cervical cancer were diagnosed in 2022, reaching almost 1793 deaths each year. While mortality is slowly decreasing, incidence remains high and constant, although it is already at an elevated level. Improvements have been observed in younger women, though significant disparities compared to the European average persist [2,3].

Cervical cancer remains preventable—its prolonged pre-clinical phase (spanning 1 to 2 decades) makes it particularly amenable to screening programs. Vaccination and screening are the primary and secondary prevention measures, respectively. Risk stratification for the progression of cervical dysplastic lesions is essential in current practice to manage high-risk cases identified through screening [4,5]. Furthermore, its pre-cancerous lesions can often be treated conservatively, without the need for hysterectomy, offering elevated cure rates [6,7].

Various combined techniques, including immunocytochemistry/immunohistochemistry, molecular biomarkers, microbiome testing methods, and artificial intelligence programs, support clinical decision-making [8,9]. To understand the mechanism of action of these risk stratification markers, it is necessary to understand the basic data on the pathophysiology of cervical cancer, human papillomavirus (HPV) infection, cervical carcinogenesis, and the natural progression of cervical intraepithelial neoplasia (CIN) [10,11,12].

Human papillomavirus (HPV) infection was identified as the primary cause of cervical cancer development [2,11,13,14]. As epidemiological studies reflect, HPV DNA is detected in nearly all cervical cancer samples (99,7%), compared to the significantly lower detection rates in controls [15,16]. While HPV is a key factor in the majority of cervical cancer cases, only a small proportion of the infected women will develop invasive cervical cancer [14,16]. This indicates the involvement of additional genetic or environmental factors in cervical carcinogenesis, which can be divided into three categories, as follows:

- The cervical immature transformation zone (a vulnerable area that facilitates HPV infection and its persistence).
- External risk factors, such as infectious agents, with HPV being the main cause, and the others acting as co-carcinogens (Human Immunodeficiency Virus, Herpes Simplex 2 Virus, Cytomegalovirus, Epstein–Barr Virus, Chlamydia trachomatis, and Trichomonas), as well as hormonal factors/anti-estrogens, nutritional factors, tobacco smoke exposure, and radiation (ionizing or ultraviolet).
- Impaired host immunity. The oncogenic potential of HPV, viral load (quantified by quantitative polymerase chain reaction, qPCR), and infection persistence are key predictors of progression from HPV infection to CIN [14,16,17].

Recent advancements in understanding the molecular mechanism behind HPV-related cervical carcinogenesis have led to the identification of potential new biomarkers that could aid in risk assessment of cervical cancer. These biomarkers might help reduce the need for biopsies. However, the real challenge for gynecologists lies in integrating these biomarkers into routine clinical practice to better assess the likelihood of cervical lesions progressing to cancer [18,19].

There are over 100 different HPV genotypes [20], with at least 42 types infecting the genital tract [21]. These types vary in their ability to cause malignant transformation, leading to the classification of HPV into low-risk (LR) and high-risk (HR) categories. The most prevalent HR types associated with cervical cancer are HPV types 16 and 18, found in more than 80% of cases. Other HR types include HPV 31, 33, 35, 39, 45, 51, 52, 56, 58, and 59. LR types (6, 11, 26, 53, 66, 67, 68, 70, 73, and 82), which can lead to dysplasia or genital warts, are rarely associated with cancer. With the development of HPV testing, it is now possible to determine whether a patient carries HR or LR types, which has proven to be a valuable tool in managing patients with low-grade Pap smear abnormalities [14,22,23].

HPV infects basal layer cells, entering through microtrauma areas created during sexual friction. The viral genome penetrates the cell membrane after shedding its capsid and is transported to the cell’s nucleus, where it establishes an episomal-type infection. At this point, the virus is present in the host’s basal cells in a small number of copies, detectable only through viral testing, and does not cause clinical or subclinical manifestations (as determined by colposcopic, cytological, and histological examinations). This phase, known as latency, is characterized by the virus’s persistence for extended periods. The subsequent steps of viral infection depend on three factors: the viral type, the infected site, and the host’s local epithelial immunity. The mechanism by which HPV infection initiates CIN lesions and cervical cancer begins with viral penetration of basal cells, followed by integration into the host genome near fragile sites and proto-oncogenes, leading to oncogene activation. When the viral DNA integrates, the circular DNA chain is cleaved, and only the fragment containing the E6, E7, and URR genes integrates. The absence of the E2 gene, which normally inhibits the expression of E6 and E7, results in their overexpression. The following steps are represented by the inhibition of the cell growth control mechanisms (E6 and E7 block tumor suppressors p53, which protects the cell from the accumulation of secondary mutations caused by DNA damage by either repairing the damage or inducing apoptosis, and retinoblastoma tumor suppressor protein (PRB), which regulates the entry into DNA synthesis at an appropriate stage of the cell cycle), resulting in cellular immortalization and malignant transformation (involving cyclins and telomerase hTERT) [24,25,26,27,28,29].

The Richard Classification of CIN, the classic histopathological classification, introduces the concept of gradual lesion progression from CIN 1 to CIN 3/in situ carcinoma to invasive carcinoma, based on the depth of the epithelial involvement and nuclear atypia [30]. Ostor’s article is a cornerstone study because it analyzed the potential evolutionary outcomes of CIN lesions according to their histological grade [31].

There are three possible outcomes for preneoplastic lesions: regression, persistence (stationary), or progression. In a study that synthetized the results of all publications from the last 40 years regarding the natural history of CIN, the following rates were recorded at 5 years: CIN 1 regresses in 80% of cases, progressing to CIN 3 in 10% and invasion in 1% of cases, while CIN 3 regresses in around 32% of cases but can progress to invasive cancer in 12%. In order to avoid overdiagnosis and overtreatment, these numbers force us to be cautious and to correctly stratify the risk of progression to CIN 3 and invasive cancer in cervical HPV infections [30,31,32,33].

Over the past several years, significant advances in understanding the molecular pathways of HPV-associated cervical carcinogenesis have revealed a growing number of potential biomarkers. Some of these may reduce the need for invasive biopsies. A rational taxonomy of these biomarkers should reflect the stage at which they emerge within the HPV-induced oncogenic cascade [34,35,36]. Accordingly, these biomarkers may be categorized as follows:

1. Cell cycle regulation markers;
2. Proliferation markers;
3. Markers of epithelial organization and differentiation;
4. Transcription factors and signaling pathway components;
5. Apoptotic markers;
6. Markers of chromosomal stability;
7. Immune recognition markers [34,35].

The paradigm of cervical cancer prevention has fundamentally shifted from cytology-based to molecular-based screening, with high-risk HPV (hrHPV) DNA testing established as the cornerstone. Major guidelines, including those from the World Health Organization (WHO), now endorse primary hrHPV testing as the preferred method for cervical screening in women aged 30 and above [37].

HPV DNA testing demonstrated higher sensitivity (97.5%) but lower specificity (85.1%) for detecting CIN3+ compared to cytology and VIA. While sensitivity was consistent across ages, specificity was highest in women under 35 (89.4%). Raising the HPV positivity cutoff from 1 pg/mL to 2 pg/mL reduced overall positivity (16.3% to 13.9%) with minimal loss in sensitivity (97.5% to 95.2%). In women under 35, a 10 pg/mL cutoff maintained high sensitivity (97.7%) while significantly improving specificity to 93.5% [38].

This high sensitivity provides a greater safety margin, allowing longer screening intervals (e.g., every 5 years) and greater reassurance against missed pre-cancerous lesions. Furthermore, as a molecular test, it is more objective than the morphological interpretation required for Pap smears. 

The primary strength of hrHPV testing is also the source of its main clinical challenge: low specificity. The test detects the presence of oncogenic HPV types but cannot distinguish between transient, clinically inconsequential infections and persistent infections that will progress to pre-cancer. In most populations, the vast majority of HPV-positive women, especially younger ones, will not have CIN2+. This low positive predictive value leads to significant overtreatment, patient anxiety, and an unsustainable burden on colposcopy services.

It is precisely this limitation—the high sensitivity but low specificity of primary HPV testing—that defines the central mission of contemporary biomarker research: to develop effective triage strategies. The biomarkers discussed in the following sections (e.g., partial genotyping, p16/Ki-67 dual stain, and methylation) are all evaluated based on their ability to effectively stratify HPV-positive women, identifying those at the highest risk who require immediate colposcopy from those who can be safely returned to surveillance.

Biomarkers have multiple applications in the evaluation and management of pre-cancerous cervical lesions. They can increase the sensitivity of cervical screening programs, allowing extended screening intervals without compromising safety and ensuring efficient triage and resource allocation. Biomarker-based stratification can help differentiate between intermediate lesions with a high probability of regression and those with a high potential for progression, allowing for surveillance and reducing the need for unnecessary invasive procedures. They also enable the monitoring of disease progression and the detection of recurrences after therapeutic interventions. The resulting risk stratification enables a more individualized clinical approach, avoiding unnecessary, invasive, and costly interventions while still allowing for early detection of silent or occult cancers. Given this expanding array of candidates, establishing a biobank containing cervical lavages, histology specimens, and blood and plasma samples has become essential to support molecular investigations. However, as the number of biomarker candidates grows, it becomes paramount to map their developmental trajectories from discovery to clinical validation. Most biomarkers will ultimately not demonstrate clinical utility and may encounter numerous pitfalls; thus, extensive testing, validation, and refinement are required before any candidate can enter clinical practice [35,39,40,41,42].

This study aims to consolidate and critically evaluate the most relevant biomarkers currently available for HPV-associated CIN lesions and cervical cancer, focusing on their clinical utility in lesion progression, screening, and risk stratification. Given the growing number of proposed biomarkers, the study seeks to provide a structured framework to guide clinicians in selecting validated, stage-specific biomarkers within the HPV-induced oncogenic pathway. Special attention is given to the clinical challenge of managing CIN2-3 lesions in young women of reproductive age, where excisional treatment may compromise future obstetric outcomes. Ultimately, the study aims to support personalized, non-invasive approaches to cervical disease management, particularly given the disease’s increasing prevalence.

## 2. Methodology

To ensure a comprehensive overview of the current landscape of cervical cancer biomarkers, a systematic literature search was conducted. The electronic databases PubMed/MEDLINE, Web of Science, and Scopus were searched for relevant articles published between 2020 and 2024. The search strategy combined key terms and Medical Subject Headings (MeSHs) related to cervical cancer and biomarkers. Core search terms included the following: (“cervical cancer” OR “cervical intraepithelial neoplasia” OR “CIN” OR “squamous intraepithelial lesion” OR “LSIL” OR “HSIL”) AND (“biomarker” OR “methylation” OR “DNA methylation” OR “S5 classifier” OR “p16” OR “Ki-67” OR “dual stain” OR “microbiome” OR “vaginal microbiota” OR “telomerase” OR “hTERT” OR “SCC-Ag” OR “viral load” OR “HPV genotyping”) AND (“screening” OR “triage” OR “diagnosis” OR “prognosis”).

The reference lists of retrieved review articles and key primary studies were also manually screened to identify additional relevant publications.

## 3. Classification of Cervical Cancer Biomarkers by Clinical Uses

The current screening already uses viral indicators, such as genotype-specific hrHPV detection. Cellular biomarkers such as p16INK4a reflect the underlying transformation process regardless of HPV type, making them attractive as single-marker strategies. In histopathology, p16INK4a staining significantly improves the reproducibility of pre-cancerous lesion classification, while in cytology, it enhances the accuracy of triaging equivocal findings. Nevertheless, more molecular biomarkers are partially implemented or under evaluation.

**Figure 1:**
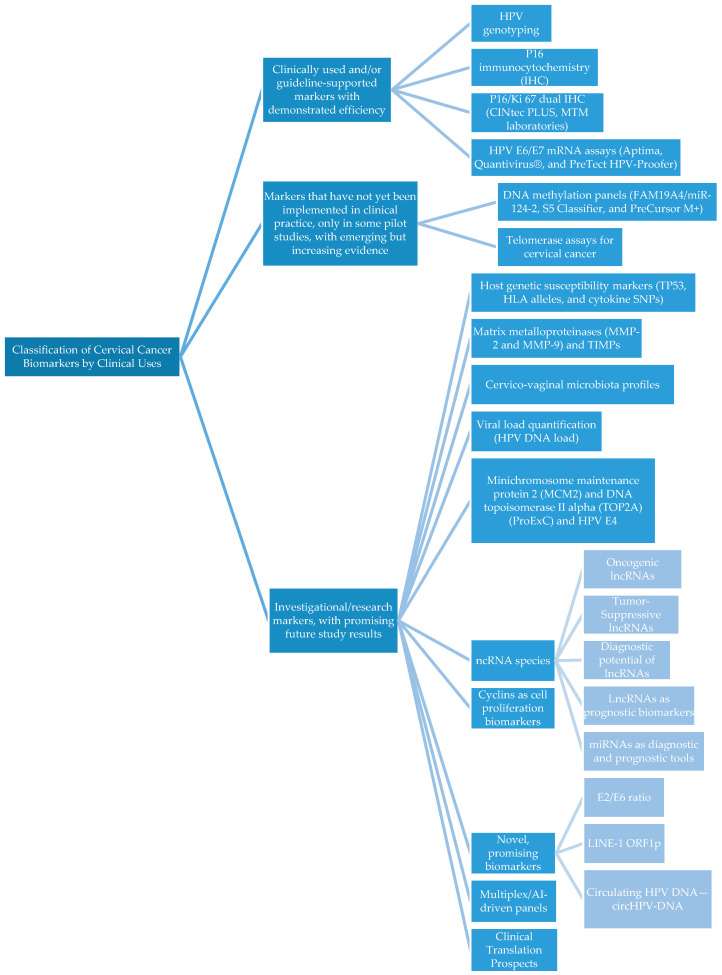
Flow chart of cervical cancer biomarkers.

According to the literature search, the biomarkers were classified into three main categories based on guideline implementation, development status, and IVD certification (Table 1 and Figure 1).

### 3.1. Clinically Used and/or Guideline-Supported Markers with Demonstrated Efficiency

#### 3.1.1. HPV Genotyping

Identifying the specific HPV genotype has become a critical test for cervical cancer screening and patient management due to its strong correlation with oncogenic potential and lesion progression [43]. HPV genotyping enables stratification of patients by viral oncogenic risk [34]. Detection of the main oncogenic type, such as HPV16 and HPV18, identifies women at the highest risk for developing high-grade cervical intraepithelial neoplasia (CIN2/3) or invasive carcinoma, supporting referral to colposcopy or enhanced surveillance. Genotype information also aids in triaging hrHPV-positive women when cytology results are ambiguous, reducing unnecessary interventions and improving resource allocation [44].

Persistent hrHPV infection can predict the progression of CIN lesions. HPV detection enables clinicians to monitor viral clearance or persistence over time, guiding follow-up intervals. Several studies demonstrated that women with persistent hrHPV infections are more likely to experience lesion progression, whereas clearance is associated with regression and lower long-term cancer risk [45].

HPV genotyping, as an implemented technique, improved triage, reduced overtreatment, and allowed personalized follow-up. Identifying high-risk infections early informs decisions on colposcopic evaluation, excisional procedures, and the frequency of surveillance. Additionally, genotype-specific data can contribute to patient counseling, vaccination strategies, and population-level screening policies. Linking specific viral types to the risk of lesion progression and persistence guides clinical management and facilitates personalized patient care.

#### 3.1.2. P16 Immunocytochemistry (IHC)

P16 IHC is a surrogate marker of transforming (oncogenic) hrHPV infection. Overexpression of p16 in cervical tissue is associated with CIN 2 + lesions and helps distinguish between lesions likely to progress versus those likely to regress [46].

Studies show that using p16 with Hematoxylin and Eosin (H&E) histology improves diagnostic reproducibility, reducing the number of misgraded or overtreated cases [47]. The Dutch study by Ebisch et al. in 2022 [48] underscores the use of p16 IHC as an adjuvant to morphology to improve triage efficiency. The authors analyzed 326 samples and concluded that adding p16 IHC led to more accurate stratification of CIN lesions, with fewer CIN 1/2 cases and more “no CIN” or CIN 3 cases, thereby avoiding overtreatment [48]. However, the literature is controversial about the use of p16 IHC alone, with the results of other studies underlining a lower specificity and more false-positive results; this supports a lack of sufficiency in triaging cases that should be referred for colposcopy or immediate treatment, with the staining being strong but not always specific enough, especially in low-grade lesion samples from younger women [47,48,49,50].

P16 immunohistochemistry (IHC) has garnered significant interest as a diagnostic and triage tool for its potential to improve cytology sensitivity and HPV genotyping specificity. The results of a study performed by Cuzick et al. on 1091 women, which included cytologic evaluation, HPV genotyping, p16 IHC analysis, and cervical biopsy, detected a significantly higher positivity rate in high-grade CIN 2+ lesions, when compared to normal or low-grade cervical ones (89.2% versus 10.2%, *p* < 0.01), with similar sensitivity and higher specificity compared to genotyping [51]. Thus, p16 appears to correlate strongly with lesion severity, reflecting both viral and host-related carcinogenic processes [52], especially in HPV 16- and 18-positive cases [53]. These observations are reinforced by Mastutik et al., who underline the diffuse p16 expression in cases with hrHPV infections and sporadic/focal or negative p16 expression in cases with hrHPV infections, thus making it a reliable surrogate marker for hrHPV activity in cervical lesions, helping to distinguish lesions with high malignant potential [54].

Khamseh et al.’s (2025) [55] study reinforces the idea that p16 can be a valuable biomarker of HPV lesion progression and oncogenic activity, reflecting viral load and genomic integration and providing a direct biological signal of transformation risk, justifying its use in diagnosis and further triage. IHC p16 expression was absent in normal cervical epithelium, had a low expression in CIN I cases (12.5%), and increased expression in CIN II (72.7%), CIN III (88.9%), and cervical carcinoma patients (90%) [55]. These differences were statistically significant (*p* < 0.01), and the expression also correlated with higher HPV-16 viral loads (*p* < 0.05) [55].

The post hoc analysis of the PROHTECT-3B trial revealed that p16 alone had higher sensitivity for CIN3+ than cytology, but lower specificity. Its adjuvant uses decrease overtreatment by reducing false-positive CIN2 diagnoses, with confidence improved in about 50% of cases [48]. Similar results were obtained in 2025 by Usta et al., who analyzed 192 cytological specimens and found that p16 positivity increased significantly with lesion severity [56].

The study by Damgaard et al. (2022) [57] on the predictive value of p16 for CIN2 regression found that low or absent p16 expression, combined with a positive HPV E4 result, indicates a cervical lesion more likely to regress. The authors underline that p16 overexpression often indicates a loss of cell cycle control under the influence of an oncogenic HPV effect, suggesting that these markers can be used to further classify CIN2 cervical lesions [57].

Song et al. (2020) [58] assessed p16 expression both as a primary screening tool and a secondary triage tool for 1197 cytology specimen slides, obtaining the same results: p16 IHC expression increased with the severity of dysplasia. The test’s sensitivity was similar to that of HPV genotyping, with twofold higher specificity. Compared with cytology, sensitivity was higher for CIN2+ lesions, with comparable specificity as a primary screening tool. As a secondary triage tool, p16 proved higher specificity after both cytology and HPV genotyping primary screening, with lower colposcopy referrals, representing a promising alternative or adjunct to both cytology and HPV testing [58].

He et al. published a flow cytometry-based study of p16 detection that included 24,100 screened women. The flow cytometry test used a monoclonal antibody clone to quantify marker expression in exfoliated cervical samples, demonstrating that the method outperforms cytology and HPV co-testing. The results consolidate p16′s position as a powerful quantitative biomarker, with improved accuracy and predictive power, reducing unnecessary colposcopies and stratifying women with low-grade lesions into high- and low-risk progression groups [59]. Another large-scale study involving 73,624 women in China, including 2557 hrHPV-positive cases, introduced p16 testing. The study demonstrated improved sensitivity and specificity, particularly among women over 50 years old, while also helping to minimize overtreatment [60].

Despite many promising findings regarding p16 use in HPV-induced cervical lesions, Miranda-Fanconi et al. (2024) reported only modest predictive value in a relatively small cohort, suggesting that p16 testing may be more suitable as part of a biomarker panel rather than as a stand-alone test [61]. The discrepancies across studies may stem from differences in sample size, which could introduce bias, or from variations in detection techniques. For instance, Usta et al., Hou et al., and Song et al. employed p16 immunocytochemistry [56,58,60], whereas Ebisch et al., Damgaard et al., and Miranda-Fanconi et al. utilized immunohistochemistry [48,57,61].

#### 3.1.3. P16/Ki 67 Dual IHC (CINtec PLUS, MTM Laboratories)

The immunocytochemistry dual staining assay for p16/ki-67 plays a central role in cervical cancer screening and triage [62].

The inactivation of p53 and the retinoblastoma tumor suppressor protein (pRb) by the E6 and E7 hrHPV oncoproteins alters several cellular pathways relevant to cell transformation and cancer development. E7 oncoprotein expression leads to pRb inactivation, the overexpression of the cyclin-dependent kinase inhibitor p16 (p16INK4a), and aberrant proliferation, as evidenced by increased Ki-67 expression. While p16 functions as a tumor suppressor by blocking G1–S phase progression, its overexpression in HPV-infected cells signals oncogenic transformation [62,63,64,65,66,67,68]. On the other hand, Ki-67 protein is a reliable marker of proliferation, and its co-expression with p16 within the same cell provides a direct indication of abnormal cell growth as a result of HPV oncogenic activity. The positive test for both biomarkers allows the triage of CIN2/3 cases [62,69].

A positive test requires dual positivity in the same cell, whereas the absence of co-expression is considered negative. The dual staining approach offers higher sensitivity than Pap cytology and provides an objective criterion for identifying patients at increased risk of developing high-grade squamous intraepithelial lesions (HSILs), who should be referred for colposcopy. Unlike p16, which may stain normal metaplastic cells, Ki-67 is restricted to proliferating cells, thereby enhancing diagnostic specificity. The method, first promoted by Christine Bergeron’s group in France, has been widely studied as a triage tool for women with ASC-US or LSIL cytology, as an alternative to HPV genotyping [63,64,65,66,70].

In Thailand, Srisuttayasathien et al. (2024) conducted a cross-sectional study, comparing p16/ki-67 dual staining with liquid-based cytology (LBC) in HPV-positive women, and found a comparable performance, with higher sensitivity but somewhat lower specificity than that reported in ATHENA, confirming the reproducibility of dual staining even in low-resource settings [71]. El-Zein et al. (2020) retrospectively analyzed 492 cervical specimens and showed that dual staining was more specific than HPV testing, particularly among younger women [72]. Similarly, Magkana et al. (2020) emphasized its specificity advantage over HPV genotyping, helping reduce unnecessary procedures [73]. The FRIDA Mexico study by White et al. found fewer unnecessary colposcopy referrals when dual staining was used in HPV16/18-positive women [74]. Additional studies reinforce these findings: Gothwal et al. (2021) showed that dual staining shows high sensitivity and specificity, especially for ASCUS and LSIL patients [75], while Luo et al. (2024) suggested its role in personalized CIN management through risk stratification [76]. Large-scale trials, including IMPACT and ATHENA, as well as real-world evidence from the Kaiser Permanente Northern California (KPNC) cohort, have validated dual staining as a robust triage tool, consistently outperforming cytology in sensitivity while providing greater specificity than HPV testing. By analyzing data from the New Technologies for Cervical Cancer Screening 2 (NTCC2) trial, Benevolo et al. (2024) combined dual staining with extended genotyping, achieving refined risk stratification that could reduce colposcopy referrals and optimize long-term surveillance intervals [77]. Thrall et al. provided a comprehensive review of the dual staining technique, highlighting technical, interpretative, and cost-related challenges [78]. Despite these limitations, multiple international studies support the use of this technique as a viable triage option, as revealed by Olivas et al. (2022), who summarized the findings from the ATHENA and IMPACT trials [79]. Dual staining seems to be superior in the screening process, especially in women under 30 years old. Secosan et al. reported higher specificity than HPV or colposcopy alone, supporting its use as a triage tool in the younger population [80]. The prospective longitudinal study by White et al. demonstrated that smoking significantly increases the risk of both p16/Ki-67 positivity and progression to CIN2+/CIN3+, underscoring the importance of lifestyle factors in risk stratification and biomarker interpretation [74]. Although CINtec PLUS has been FDA-approved in the United States since 2020, interesting findings published by Ying Li et al. (2022) [81] warrant further investigation. In their comparison of the CINtec PLUS and Dalton assays, the authors concluded that Dalton may offer a superior performance, with lower false-positive rates for identifying high-grade CIN [81]. A limitation of dual staining, highlighted by Stoler et al. in the Onclarity trial, is that although dual staining and HPV genotyping show comparable performance in detecting CIN3+ lesions, genotyping offers broader applicability, particularly because it is compatible with self-sampling [82]. Moreover, Macios and Nowakowski emphasized that false-negative results in dual staining are often linked to interpretative challenges, insufficient reader training, and methodological inconsistencies [83].

However, a real-life study showed that incorporating p16/Ki67 dual staining as a triage method for women who test positive for hrHPV—using limited genotyping—provides superior diagnostic accuracy for identifying cervical pre-cancer compared to cytology-based triage within primary HPV screening programs. The markedly higher specificity of the dual stain approach suggests a substantial potential to reduce the number of unnecessary colposcopies, both among HPV16/18-positive women and those infected with other high-risk HPV genotypes. Therefore, implementing p16/Ki67 dual staining into cervical screening algorithms could significantly enhance secondary prevention strategies for cervical cancer [84].

Harper et al. (2025) showed that numerous studies across various geographic regions demonstrated high sensitivity and specificity of p16/Ki67 dual staining when used for the triage of HPV-positive women [85]. The p16/Ki67 dual staining method was validated in the large prospective PALMS study (27,349 women), showing significantly higher sensitivity than cytology for detecting CIN2+ (86.7% versus 68.5%, *p* < 0.001), as well as comparable specificity (95.2% versus 95.4%, *p* = 0.15) [86]. In McMenamin’s study, the kappa values for agreement between p16/Ki-67 dual stain reviewers fell within the “very good” range [87]. In contrast, the summary kappa values reported in other studies indicated “good” inter-reader agreement, ranging from 0.61 to 0.71 [88,89,90]. Agreement improves when more dual-stained cells are present, while slides with only a single positive cell introduce greater uncertainty. Variability also increases with weak p16 staining, poor cell morphology, or background artifacts. Although basic training ensures reasonable consistency, additional expert-led training and reviewing of ambiguous cases are essential to enhance accuracy and minimize inter-laboratory differences [91].

#### 3.1.4. HPV E6/E7 mRNA Assays (Aptima, Quantivirus^®^, and PreTect HPV-Proofer)

Detection of E6/E7 messenger RNA indicates viral integration into the host genome and the initiation of oncogenic transformation, allowing for the identification of both hrHPV strains and the resulting cervical lesions. The literature highlights its strong correlation with CIN2/3 lesions. In some countries, the HPV E6/E7 mRNA test is used alongside cytology to complement primary screening based on HPV DNA detection [92,93].

Derbie et al. (2020) [94] conducted a systematic review of 29 studies including 23,576 women aged 15–85 years with varying cervical pathologies. All participants underwent HPV E6/E7 mRNA testing following positive cytology or HPV DNA results. Among the available assays, the Aptima test has been the most extensively investigated. Seven of the included studies evaluated the role of E6/E7 mRNA testing in triaging women with abnormal Pap smears or HPV-positive results, while eight studies compared its diagnostic accuracy with HPV DNA testing. E6/E7 mRNA-based assays differ as follows: the PreTect HPV-Proofer targets only five high-risk genotypes (16, 18, 31, 33, and 47), yielding higher specificity, whereas Aptima and Quantivirus cover a broader range of genotypes, offering greater sensitivity but lower specificity. This type of test provides more clinically meaningful information, as it reflects viral oncogenic activity and correlates more strongly with lesion severity. It helps identify women at higher risk of cervical cancer while allowing longer follow-up intervals for those testing negative, with a negative predictive value ranging from 77% to 99.8% [92,93,94,95].

Downham et al. conducted a meta-analysis of 22 studies, revealing that E6/7 oncoprotein testing has high specificity across populations (>82%) but moderate sensitivity (46.9–75.5%) depending on HPV risk groups. Specificity was notably lower in HPV 16/18-positive women compared with other groups. Testing E6/E7 oncoprotein seems promising for triaging HPV-positive women, although it demonstrated a lower sensitivity, likely due to limited viral integration in non-progressing pre-cancerous lesions. Further longitudinal studies are needed to validate its predictive role [96]. Singini et al. found that HPV 16/18 E6/E7 antibodies showed high specificity but low sensitivity for detecting CIN2+ lesions, suggesting limited applicability as a primary diagnostic tool but possibility as a surrogate marker of immune response in advanced disease [97]. A 2024 meta-analysis by Xu et al., which included 2224 women, confirmed the test’s high sensitivity for CIN2+ detection and its ability to reduce missed diagnoses, while its specificity was relatively low. This low specificity raises concern about potential overdiagnosis and unnecessary interventions. An important limitation of this meta-analysis is that it did not directly compare this test with other screening modalities, thereby perhaps limiting essential comparative aspects [98]. Observational studies have provided additional insights into clinical application. Jin et al. (2023) demonstrated its value for triaging colposcopy referrals in women with ASC-US cytology and in postmenopausal women positive for E6/E7 mRNA [99]. Zhang J et al. (2024) confirmed an age-dependent performance, with higher efficiency in women aged between 35 and 44 years old and those between 55 and 64 years old, but less reliable in peri-menopausal women [100]. Moreover, Liu Y et al. (2023) and Gupta et al. (2022) reported superior specificity of E6/E7 mRNA testing compared to DNA HPV assays for CIN2+ detection, suggesting its potential use in primary screening and immediate colposcopy referral for HPV-positive patients with negative cytology [101,102]. Moreover, Liu et al. (2020) and Ren et al. (2019) [103,104] proposed a quantitative assay of the E6/E7 copy number to better differentiate low-grade from high-grade lesions, although the optimal cutoff value remains to be established. E6/E7 mRNA testing achieves high sensitivity and a low rate of missed CIN2+ lesions, but its moderate specificity increases the risk of overdiagnosis and unnecessary colposcopy referrals, limiting its role [103,104]. Its prognostic utility, however, seems promising, as its negative expression correlates with higher rates of lesion regression.

### 3.2. Markers That Have Not Yet Been Implemented in Clinical Practice, Only in Some Pilot Studies, with Emerging but Increasing Evidence

#### 3.2.1. DNA Methylation Panels (*FAM19A4/miR-124-2*, S5 Classifier, and PreCursor M+)

hrHPV detection, recommended by the WHO for cervical cancer screening, has proven more sensitive than cytology in detecting CIN lesions. However, its specificity remains limited, as the majority of infections are transient, with around 80% clearing the virus within one year of acquisition. Thus, this underlies the need for more specific assays for the triage of HPV-positive women who need further colposcopy referral, as only about 20% of hrHPV-related lesions actually progress. The process of aberrant DNA methylation occurs during pre-cancerous progression, thus permitting the identification of cases with HPV infection that will progress to cancer. DNA methylation is an epigenetic modification that inactivates host tumor suppressor genes and serves as an early signal of malignant transformation. Methylation levels rise with increasing CIN severity, providing a molecular signal of malignant potential. Unlike Pap cytology or dual staining, methylation assays can be performed on either clinician-collected or self-collected samples, simplifying logistics and reducing reliance on observer interpretation. However, costs limit their availability in low-resource settings. Methylation assays have higher specificity but somewhat lower sensitivity than HPV DNA screening, making them particularly valuable for triage rather than primary screening. Candidate genes frequently evaluated for hypermethylation include *CADM1*, *MAL*, *miR-124*, *FAM19A4*, *PAX1*, and *SOX1* [105,106,107]. A meta-analysis of 43 studies by Chan KKl et al. reported pooled sensitivity and specificity of 63.2% and 75.9%, respectively, for methylation testing, though the optimal gene panel is still debated. Several commercial assays are currently under development or already in clinical use: the QIAsure Methylation test (targeting *FAM19A4* and *miR124-2*), S5-classifier (including *EPB41L3* and HPV16-*L1*, HPV16-*L2*, HPV18-*L2*, HPV31-*L1*, and HPV33-*L2*), and GynTect^®^ (including *ASTN1*, *DLX1*, *ITGA4*, *RXFP3*, *SOX17*, and *ZNF671*). Clinical studies suggest potential prognostic applications as well. For example, women who test negative for *PAX1* methylation exhibit a significantly lower risk of subsequently developing high-grade lesions than those with negative cytology or even HPV16/18 positivity. This supports the notion that HPV-positive but *PAX1*-negative women could safely undergo extended surveillance intervals [106,108].

Accumulating evidence supports the role of DNA methylation assays as complementary tools to hrHPV testing in cervical cancer screening. A systematic review and meta-analysis by Salta et al., including 23 studies, reported pooled sensitivity ranging from 0.68 to 0.78 for CIN2+ and CIN3+ detection, with specificities of 0.75 and 0.74, respectively. The most frequently evaluated methylation markers were *CADM1*, *FAM19A4*, *MAL*, and *miR124-2*. Despite these encouraging results, significant heterogeneity in study design, populations, and cutoff definitions remains an obstacle to translation into routine implementation [108]. A larger review and meta-analysis of over 16,000 women across 43 studies, conducted by Kelly et al., noted that DNA methylation detection achieves a higher specificity than cytology for ASC-US + lesions, and a higher sensitivity than HPV 16/18 genotyping, with a reported positive predictive value (PPV) of 53% for CIN2+ and 35% for CIN3+. The assay offers notable advantages, including automation, reduced subjectivity, and compatibility with self-sampling approaches [105]. Several host-gene markers and panels have been validated in both prospective and retrospective settings. *PAX1* and *ZNF582* methylation are strongly correlated with lesion severity and p16/ki-67 expression and may act as triggers in the progression from HPV infection to CIN3+ [76]. The GynTect^®^ assay has shown high specificity and strong negative predictive value, thereby reducing unnecessary colposcopies [109,110,111,112]. *FAM19A4/miR124-2* has been validated extensively, including in the POBASCAM 14-year follow-up, where a negative result was associated with a low long-term cervical cancer risk among HPV-positive women, outperforming cytology [113]. Other gene panels have also demonstrated clinical feasibility. *ASCL1/LHX8* methylation panel was validated in the Dutch IMPROVE trial, yielding 76.9% sensitivity and 74.5% specificity for CIN3+, comparable to HPV16/18 genotyping [114]. The S5 methylation classifier, combining both viral and host genes, showed superior sensitivity compared to cytology and genotyping, with the advantage of being suitable for self-sampling and reflex testing [115]. Fackler et al. (2024) proposed a five-gene panel comprising *FMN2*, *EDNRB*, *ZFN671*, *TBXT*, and *MOS*, showing robust sensitivity and specificity across cohorts from Vietnam, South Africa, and the United States [109]. The same year, Vieira-Baptista et al. confirmed the feasibility of GynTect^®^ in organized screening, detecting 78% of CIN3+ cases while reducing colposcopy referrals by 75% [110]. Ren et al. supported the single ZFN671 methylation marker performance, suggesting its cost-efficiency for triage [111], while Chen et al. optimized a three-gene methylation panel, including JAM3, PCDHGB7, and SORCS1, that outperformed cytology and HPV 16/18 genotyping in CIN3+ detection [112]. Importantly, Louvanto et al. (2024) [107] investigated methylation patterns in HPV-vaccinated women. The study showed lower methylation levels in HSIL associated with non-HPV16/18 genotypes, suggesting limited progression potential. This observation underscores the need for tailored management strategies in vaccinated cohorts, as the predictive value of methylation testing may be lower than in unvaccinated populations [107]. 

#### 3.2.2. SCC Antigen (SCC-Ag) in the Diagnosis and Prognosis of Cervical Cancer

Squamous cell carcinoma antigen (SCC-Ag, also called SCCA, with isoforms SCCA1/SERPINB3 and SCCA2/SERPINB4) is a glycoprotein first isolated from squamous carcinomas and is widely studied as a serum tumor marker in cervical squamous cell carcinoma [116]. SCC-Ag is not disease-specific, as its expression can be raised in other squamous malignancies and some benign conditions; however, in cervical cancer, it correlates with tumor burden and biologic aggressiveness and has practical clinical applications in pretreatment risk stratification, monitoring treatment response, and surveillance for recurrence [117].

Serum SCC-Ag levels increase with FIGO stage, primary tumor size, nodal involvement, and other adverse pathologic features [118]. Several studies have shown that higher pretherapy SCC-Ag levels are associated with more advanced disease and a greater likelihood of lymph node metastasis; therefore, SCC-Ag can complement imaging and clinical staging by identifying patients at higher risk who may benefit from intensified staging or tailored treatment planning [119,120,121]. However, sensitivity is stage-dependent (lower in early FIGO I disease, and higher in advanced stages), so SCC-Ag should not be used as a sole screening tool [117].

Elevated SCC-Ag pretreatment is repeatedly associated with worse outcomes (lower disease-free and overall survival) across cohorts treated with surgery or definitive chemoradiotherapy [122]. The magnitude of SCC-Ag and its failure to normalize after therapy have been incorporated into prognostics, predicting distant recurrence and survival; patients with high pre- or persistently elevated post-treatment SCC-Ag have higher rates of local, regional, and distant failure. These properties make SCC-Ag a useful biomarker for risk stratification and for informing decisions such as the extent of surgery, need for adjuvant therapy, or more intensive surveillance [121].

Serial SCC-Ag measurements during and after treatment reflect tumor response: most responsive tumors show marked declines in SCC-Ag during chemoradiation, while persistently high or rising levels predict residual disease or early recurrence—often preceding clinical or radiological detection by months [121,123]. Recent findings support SCC-Ag as a cost-effective adjunct to follow-up algorithms for selecting patients for imaging or earlier intervention, using predefined cutoffs to trigger further evaluation [122,124]. Nonetheless, non-cancer causes of SCC-Ag elevation and transient post-treatment fluctuations require cautious interpretation [117]; this implies some limitations, along with variability in assay cutoffs between laboratories and lower sensitivity in early disease [125]. However, a newly published study refines optimal cutoffs, measurement timing, and the combination of SCC-Ag with molecular markers (HPV metrics, methylation, ctDNA) to improve early detection of recurrence and personalize follow-up [126].

#### 3.2.3. Telomerase Assays for Cervical Cancer (The TRAPeze^®^ Assay, Particularly with Versions Such as the TRAPeze^®^ RT Kit, and the Telomeric Repeat Amplification Protocol (TRAP))

Telomerase is a ribonucleoprotein enzyme essential for telomere maintenance and cellular immortalization. While its RNA component (hTR/TERC) is constitutively expressed, the transcription of the catalytic subunit *hTERT* is tightly regulated and normally repressed in differentiated cells. The activation of telomerase through *hTERT* expression is a hallmark of malignant transformation. It has been consistently detected in exfoliated cervical cells, CIN3 lesions, and more than 90% of cervical carcinomas [127,128].

The cloning of the *hTERT* promoter enabled detailed analyses of its regulation. Oncogenic transcription factors such as *c-Myc* activate *hTERT* expression, whereas repressors, including *WT1*, *Mad1*, *Mxi1*, *BRCA1*, and *p53*, can suppress its transcription [129,130,131,132,133,134]. Epigenetic regulation also plays a critical role: the *hTERT* promoter is CpG-rich and subject to methylation, which correlates with transcriptional activity in tumor cells [129,135,136].

HPV oncoproteins further influence telomerase regulation. HPV16/18 *E6* upregulates *hTERT* transcription via interactions with E6TP1 and E6AP, contributing to the immortalization of keratinocytes. Conversely, the viral E2 protein can repress *hTERT* by binding directly to its promoter, though this repression is frequently lost during viral genome integration in cervical cancer [137,138].

Recent work has expanded our understanding of telomerase biology. *hTERT* is now known to exert non-canonical functions in DNA repair, chromatin remodeling, and regulation of oxidative stress, further supporting tumor cell survival [139]. Importantly, *hTERT* promoter mutations (C228T and C250T), among the most frequent non-coding mutations in human cancer, have been described as strong prognostic biomarkers in glioblastoma, melanoma, and urothelial carcinoma [140], though they occur less frequently.

Elevated *hTERT* expression and telomerase activity correlate with HPV status, invasive potential, and poor prognosis in cervical cancer [29]. At the same time, novel therapeutic approaches—including CRISPR/Cas9-mediated disruption of *hTERT* and small-molecule telomerase inhibitors—are being actively investigated as strategies to target the immortalization machinery of cancer cells [141].

Taken together, *hTERT* serves not only as a marker of immortalization but also as a key molecular hub integrating oncogenic signaling, viral infection, and epigenetic control in cervical carcinogenesis. Its dual role as a biomarker and therapeutic target continues to attract significant research interest.

Despite the compelling mechanistic rationale for telomerase reactivation in cervical carcinogenesis, it is important to note that all current telomerase assays, including TRAP-based methods and *hTERT* detection, are strictly in the research and development phase. There are currently no FDA-approved or CE-IVD-certified telomerase-based tests for cervical cancer screening in routine clinical use. Thus, while its translational potential is significant, the clinical utility of telomerase for cervical screening triage remains investigational.

### 3.3. Investigational/Research Markers with Promising Future Study Results

#### 3.3.1. Host Genetic Susceptibility Markers (TP53, HLA Alleles, and Cytokine SNPs)

Although persistent HPV infection is the main driver of cervical carcinogenesis, genetic predisposition may influence progression to HSIL and invasive disease. This disparity suggests that host genetic background plays an important role in determining susceptibility, influencing immune responses, viral clearance, and genomic stability. Several germline polymorphisms in immunity, DNA repair, detoxification, and folate metabolism genes have been studied in relation to cervical cancer risk.

Candidate gene studies have explored variants in tumor suppressor and DNA repair genes, including *TP53* [142], *MDM2* [143], *ATM* [144], *BRIP1* [145], *CDKN1A* [146], *CDKN2A* [147], *FANCA*, *FANCC*, *FANCL* [148], *XRCC1* [149], and *XRCC3* [150]. Immune-related genes have also been implicated, such as *CD83* [151], *CTLA4* [152], and *CARD8* [153], as well as cytokine genes encoding *TNF-α* [154], *ILs* [155], *TGFB1* [156], and *IFNG* [157] (Table 2). Despite numerous reports, most associations failed replication in large case–control or meta-analysis datasets, with the exception of certain HLA alleles [158]. The *TP53 Arg72Pro* variant remains debated [118].

Genome-wide association study (GWASs)-type approaches have provided stronger evidence for genetic susceptibility. The first GWAS in a Swedish cohort confirmed known HLA associations (e.g., *HLA-B07:02*, *HLA-DRB1*13:01-*DQA101:03-DQB1*06:03, *HLA-DRB115:01-DQB1*06:02) and identified novel MHC loci: *rs9272143* (between *HLA-DRB1* and *HLA-DQA1*), *rs2516448* (near *MICA*), and *rs3117027* (at *HLA-DPB2*) [163,164].

An East Asian GWAS discovered *rs59661306* on 5q within *ARRDC3*, a tumor suppressor gene, though this finding has not been replicated in Europeans [165]. A large trans-ethnic meta-analysis (Estonian, UK, Finnish, and Japanese cohorts) identified five loci linked to invasive cervical cancer, including a novel association at *LINC00339/CDC42* (*rs2268177*) on 1p36. Additional signals were found at DAPL1 (*rs12611652)* on 2q24 for cervical dysplasia and *CD70* (*rs425787*) on 19p13 in joint analyses [166].

Rare variant sequencing has also revealed high-impact risk alleles. An Icelandic study associated *PTPN14* loss-of-function variants with a markedly increased risk of cervical cancer (OR 12.7, *p* = 1.6 × 10^−4^) and earlier age at onset [132]. Since *PTPN14* encodes a phosphatase targeted by HPV E7 and regulates the Hippo–YAP pathway, germline mutations may enhance oncogenic potential [167].

#### 3.3.2. Matrix Metalloproteinases (MMP-2 and MMP-9) and TIMPs

Research on HPV-associated cervical lesions and neoplasia has increasingly focused on the role of matrix metalloproteinases (MMPs). These zinc-dependent endopeptidases catalyze the degradation of extracellular matrix components, a process fundamental to tumor invasion. Their proteolytic activity is endogenously regulated by tissue inhibitors of metalloproteinases (TIMPs), with the broad-spectrum inhibitors TIMP-1 and TIMP-2 capable of suppressing all known members of the approximately 23 MMP isoforms. In the pathogenesis of HPV-related cervical lesions, MMPs facilitate invasion and metastasis by mediating the dissolution of the basement membrane [168,169,170,171].

Increased levels of MMP-2 and MMP-9 correlate with progression from CIN to invasive carcinoma, reduced overall survival, and shorter recurrence-free survival [172]. MMP-2, in particular, serves as a strong biomarker, with > 90% accuracy for identifying invasive lesions, showing progressive upregulation with lesion severity, while TIMP-2 expression remains stable. Consequently, an altered MMP-2/TIMP-2 ratio reflects a highly aggressive tumor microenvironment [168].

MMP-7 has been associated with cell proliferation, migration, and invasion, acting in an oncogenic-like manner. Zhu et al. (2018) proposed its potential clinical utility as a biomarker for HPV-induced cervical carcinoma [173].

In contrast, some studies have linked MMP-9 expression to recurrence risk, indicating its potential as a prognostic marker; however, these findings remain inconsistent and lack broad validation in the literature [174]. Overall, MMP/TIMP profiling, when combined with molecular and epigenetic biomarkers, may enhance risk stratification and guide personalized management of cervical dysplasia. Their integration into predictive algorithms could be particularly valuable in cases with equivocal biopsy findings or in monitoring patients with persistent HPV infection. However, the role of metalloproteinases as reliable biomarkers in HPV-induced lesions remains controversial and warrants further investigation [168,169,170,171,175].

#### 3.3.3. Cervico-Vaginal Microbiota Profiles (Community State Types, and Lactobacillus Dominance Versus Dysbiosis)

Microbiome diversity is considerably greater than genomic variation: while human genomes are 99.9% identical, microbiota composition can differ by 80–90% in regions such as the palmar region or the intestine [176]. This significant variation highlights the potential of microbiome-based approaches in personalized medicine, shifting the focus from the relatively static human genome to the dynamic genetic profiles of colonizing microorganisms [176,177,178,179]. In the female reproductive tract, *Lactobacillus* species offer key protection through “competitive exclusion,” preventing pathogen adhesion to the vaginal epithelium. Although over 120 species have been identified, vaginal communities are typically dominated by 1 or 2 species [180]. A lactobacillus-dominated vaginal microbiota supports health by producing lactic acid, bactericidal substances, and hydrogen peroxide and by blocking pathogen adhesion [181]. This protective role is critical given that cervical cancer, primarily caused by persistent infection with hrHPV types (e.g., HPV-16 and HPV-18), remains a major health burden. While most infections are cleared, persistent HPV evades immune responses by altering Toll-like receptor (TLR) signaling and producing E6/E7 proteins that suppress antiviral cytokines, including IFN-α and IFN-β [182,183,184].

Dysbiosis, characterized by reduced *Lactobacillus* and increased anaerobes such as *Gardnerella* and *Prevotella*, is linked to persistent HPV infection and cervical intraepithelial neoplasia (CIN). *Lactobacillus* species modulate immunity by reducing pro-inflammatory cytokines (IL-1β, IL-6, IL-8, and IL-10) and enhancing anti-inflammatory ones (IL-2 and IL-7), creating conditions that hinder viral persistence [185,186]. Certain strains, especially *L. gasseri*, can modulate epithelial immune responses and suppress HPV-positive cervical cancer cell growth without triggering inflammation [187,188,189]. The shift from a Lactobacillus-dominant community to dysbiosis alters cytokine profiles, reduces lactic acid production, and increases inflammation, contributing to HPV persistence and progression to intracervical neoplasia (CIN) and cervical cancer [184,189,190]. Vaginal flora can be classified into five community state types (CSTs): CST I—*L. crispatus*-dominant: most stable and protective (pH 3.8–4.4); CST II—*L. gasseri*-dominant: protective but less stable than CST I; CST III—*L. iners*-dominant: transitional and less protective; CST IV—Low Lactobacillus, high anaerobes (e.g., Gardnerella, Atopobium, and Prevotella): associated with BV; high pH: subdivided into IV-A and IV-B; and CST V—*L. jensenii*-dominant: protective but less common [184,185,187].

The cervico-vaginal microbiome represents a source of potential biomarkers, with specific taxa showing consistent associations with cervical oncogenesis and HPV persistence. Notably, a decreased abundance of the protective *L. crispatus*, an increased abundance of the more transient *L. iners*, and the enrichment of anaerobe species such as *Gardnerella or Prevotella* are consistently associated with a higher risk of cervical intraepithelial neoplasia [191,192,193].

The profound implications of these findings for future diagnostics underscore the importance of continued research to move these promising markers from association to clinical application.

#### 3.3.4. Viral Load Quantification (HPV DNA Load)—The Seegene Anyplex System and Roche Cobas

The correlation between HPV viral load and cervical lesion severity remains insufficiently understood, and the clinical significance of viral load in both detection and treatment continues to be debated. Considerable controversy persists over the utility of viral load as a biomarker for assessing and diagnosing cervical disease. Current methodologies for quantifying HPV viral load include quantitative polymerase chain reaction (qPCR), Hybrid capture 2 (HC2), and in situ hybridization (ISH). However, heterogeneity in reporting metrics poses challenges for cross-study comparison: qPCR results are often expressed as copies per cell, per volume, or per host genome, whereas HC2 typically reports values as relative light units per cutoff (RLU/CO). In some cases, integrated optical density has also been employed. Importantly, there is no consensus regarding a standardized cutoff threshold to define “low”, “medium”, or “high” viral loads [194,195,196,197,198,199,200].

A systematic review by Fobian et al. (2024) [194] demonstrated that elevated HPV viral load is generally associated with increased disease severity and poorer clinical outcomes. Current evidence is most consistent for overall HPV viral load and HPV 16-specific viral load, with both showing a positive correlation with lesion grade. In efforts to improve the diagnostic accuracy of second-line cervical screening, there is growing interest in integrating viral load quantification with other molecular markers, such as co-infection status. HPV viral load assessment may also provide insight into infection persistence, as higher viral loads are more frequently associated with chronic infections that are more likely to progress. Longitudinal studies further suggest that dynamic changes in viral load correlated significantly with the risk of developing CIN 2+ lesions, particularly when considered in relation to specific genotypes [194,195,196].

The implications of the HPV genotype are critical, as viral load patterns differ across subtypes. For example, viral loads of genotypes related to HPV 16, comprising 52 and 58, tend to increase with disease progression, whereas viral loads of subtypes 45 and 59, related to HPV 18, show relatively stable patterns. Viral integration into the host genome further complicates interpretation, as integrated viral DNA may drive oncogenic transformation even when measurable viral load appears low, potentially leading to an underestimation of disease severity and delayed clinical intervention. Moreover, age-related differences have been reported: the correlation between viral load and lesion grade is stronger in women over 30 years old compared with younger women. The impact of multiple HPV infections and co-infection remains controversial. Some studies suggest that co-infection may potentiate the risk of progression to high-grade lesions, whereas others report no significant difference in cervical cancer risk between women with single versus multiple infections. Collectively, these findings indicate that high-risk HPV viral load influences cervical disease development to varying extents, depending on genotype, infection dynamics, and host factors [195,196,199].

Efforts have been made to establish clinically relevant cutoff values for viral load. Liu et al. proposed that colposcopy should be performed when a patient has a viral load exceeding 10 RLU/CO, while Pap smear cytology should be prioritized for intermediate values (>1 to <10 RLU/CO) to optimize sensitivity, specificity, and referral rates [195,200,201]. Similarly, Lorincz et al. classified hrHPV DNA viral load into three categories: low (1–99.99 RLU/CO), moderate (100–999 RLU/CO), and high (>1000 RLU/CO). Despite variability, most studies employing HC2 have adopted similar groupings, defining a “low” viral load as 1–10 RLU/CO, “medium” as 11–100 RLU/CO, and “high” as 101–1000 RLU/CO [195,197,200].

#### 3.3.5. Minichromosome Maintenance Protein 2 *(MCM2*) and DNA Topoisomerase II Alpha (*TOP2A*) (ProExC) and HPV E4

Recent research has shown that *MCM2* and *TOP2A* are upregulated in cells exhibiting aberrant S-phase activity, including those transformed by HPV. Their expression is strongly correlated with increased levels of the viral oncoproteins E6 and E7 [202,203,204,205,206]. MCM proteins (consisting of MCM2, MCM5, MCM6, and MCM7) are highly conserved DNA-binding factors essential for replication licensing. MCM2 is expressed only in the normal basal proliferating cervical epithelium, and its dysregulation disrupts DNA replication processes [44,206,207].

The ProExC^TTM^ (BD Diagnostics-Tripath, Burlington, NC, USA) assay utilizes a monoclonal antibody combination targeting both MCM2 and TOP2A to detect abnormal proliferative activity [203,205,206]. This nuclear staining technique highlights dysplastic cells; however, false-positive results may occur in certain contexts, including normal basal and parabasal cells in atrophic epithelium, or in metaplastic glandular and tubal epithelium [203]. Compared with p16 immunostaining, ProExC^TM^ demonstrates greater sensitivity for identifying women with LSIL. Nevertheless, its specificity for accurately distinguishing LSIL is comparatively lower [63,202,204,207].

#### 3.3.6. ncRNA Species

While infection with high-risk human papillomavirus (hrHPV) is a necessary precondition for cervical cancer, it alone is insufficient to drive carcinogenesis. The progression to malignancy is critically influenced by host factors, including individual genetic variations and epigenetic modifications. Epigenetic mechanisms—such as DNA methylation, histone modification, and the action of non-coding RNAs (ncRNAs)—regulate gene expression without altering the DNA sequence itself. Non-coding RNAs (ncRNAs) are functionally versatile RNA molecules that regulate gene expression without being translated into proteins. In the context of cervical cancer, research has primarily focused on three key categories: long non-coding RNA (lncRNA), microRNA (miRNA), and circular RNAs (circRNAs).

Among these, ncRNAs, which are abundant in the genome, are categorized by size into small ncRNAs and lncRNAs exceeding 200 nucleotides [208]. LncRNAs are further classified based on their genomic context relative to protein-coding genes and function through both cis- and trans-regulatory mechanisms. Advances in high-throughput sequencing have unveiled the critical roles of ncRNAs in diverse biological processes, including development, proliferation, and DNA damage repair [209,210]. Consequently, the dysregulation of lncRNAs has been strongly linked to the pathogenesis of various cancers and other diseases, highlighting their immense potential as diagnostic biomarkers and therapeutic targets [211].

##### Oncogenic lncRNAs in Cervical Cancer

Numerous lncRNAs function as oncogenes in cervical cancer, driving tumor progression through diverse mechanisms (Table 3). Key players include H19, which promotes proliferation by sponging miR-143-3p to upregulate *SIRT1*, and *MALAT1*, which facilitates invasion by epigenetically silencing *miR-124* and thereby disinhibiting GRB2 [212,213]. HOTAIR enhances metastasis by co-activating SRF, activating STAT3-mediated transcription, and sponging miR-148a to upregulate *HLA-G* [214]. CCAT1 stimulates proliferation and invasion by sponging miR-181a-5p and activating Wnt/β-catenin signaling, while XIST stabilizes the Fus oncoprotein by sequestering miR-200a [215,216]. Additionally, SNHG family members (SNHG14, SNHG12, SNHG16, and SNHG20) contribute to oncogenesis by regulating distinct pathways including the miR-206/YWHAZ, miR-125b/STAT3, miR-216-5p/ZEB1, and miR-140-5p/ADAM10-MEK/ERK axes [217].

##### Tumor-Suppressive lncRNAs in Cervical Cancer

In contrast to oncogenic lncRNAs, only a limited number function as tumor suppressors in cervical cancer. MEG3 induces apoptosis by binding to phospho-STAT3 and promoting its ubiquitination, while *GAS5* is frequently silenced epigenetically and functions by sponging miR-196a and miR-205 to upregulate *FOXO1* and *PTEN* [218,219]. Similarly, STXBP5-AS1 and TUSC8 also inhibit proliferation and invasion by targeting PTEN [220,221].

The lncRNA XLOC_010588 is a significant prognostic marker, negatively correlating with FIGO stage and serving as an independent predictor of overall and progression-free survival. It suppresses tumor growth by directly interacting with c-Myc and reducing its expression [222].

##### Diagnostic Potential of lncRNAs in Cervical Cancer

With rising incidence in younger populations and poor overall survival for advanced/recurrent disease, there is a pressing need for more specific and sensitive biomarkers. Long non-coding RNAs (lncRNAs) represent promising non-invasive diagnostic tools for cervical cancer. Notably, serum levels of lncRNA GIHCG are significantly elevated in cervical cancer patients. ROC curve analysis demonstrates its strong diagnostic capability, with 88.75% sensitivity and 87.50% specificity in distinguishing patients from healthy controls [223]. Similarly, PVT1 shows markedly higher serum expression in cervical cancer groups compared to healthy individuals, indicating its utility as both a diagnostic and prognostic biomarker [224]. Despite these promising findings, several challenges must be addressed before clinical implementation. Further research is essential to identify lncRNAs with optimal specificity and sensitivity for cervical cancer diagnosis.

##### LncRNAs as Prognostic Biomarkers in Cervical Cancer

Numerous long non-coding RNAs (lncRNAs) demonstrate significant prognostic value in cervical cancer, with their expression levels or methylation status strongly correlating with clinical outcomes. The aberrant expression of lncRNAs such as *LINC00511*, *CERNA2*, *GHET1*, and *SOX21-AS1* has been consistently associated with advanced disease stage, lymph node metastasis, and poorer overall survival [225,226,227,228]. Furthermore, the methylation status of specific lncRNAs, including *MEG3*, serves as a valuable prognostic indicator, with elevated methylation levels predicting aggressive tumor behavior and disease progression [229]. Additional lncRNAs, such as AC126474 and C5orf66-AS1, show promise in predicting metastatic potential [230]. The stability and detectability of these molecules in clinical samples underscore their potential utility as non-invasive prognostic tools for cervical cancer management.

##### miRNAs as Diagnostic and Prognostic Tools in Cervical Cancer

MicroRNAs (miRNAs) have emerged as promising biomarkers for cervical cancer (CC) diagnosis and prognosis due to their stable presence in bodily fluids (cervical swabs, serum, plasma, and urine) and distinct expression patterns in cancerous versus healthy tissues. Their dysregulation reflects key aspects of disease progression, offering clinical potential for non-invasive detection and risk stratification [231].

The discovery that circulating miRNAs are protected within exosomes and vesicles has established their potential as stable, non-invasive biomarkers for cervical cancer. Diagnostic panels can leverage both the increased expression of specific miRNAs—such as *miR-21*, *miR-27a*, *miR-34a*, and *miR-196a*, which are highly expressed in cervical squamous cell carcinoma (SCC)—and the aberrant hypermethylation of others. For instance, the hypermethylation of *miR-124*, detectable via methylation-specific PCR (MSP), serves as a diagnostic indicator [232,233]. Furthermore, hypermethylation of *miR-203* and *miR-375* is associated with HPV-positive high-grade dysplasia, making them potential indicators of pre-cancerous lesions [234]. Analyzing a combination of miRNA expression and methylation patterns provides a powerful approach for diagnosing cervical cancer across its various stages.

Cervical swabs are a promising biosource for prognostic and diagnostic biomarkers in cervical cancer (CC), particularly given the presence of differentially expressed microRNAs (miRNAs). Investigations into these fluid-derived miRNAs reveal distinct expression patterns associated with disease progression. For instance, *miR-432* shows significant downregulation in cervical mucus from CINII/III lesions compared to normal samples [235]. Conversely, a panel of miRNAs—including miR-26b-5p, miR-142-3p, miR-143-3p, miR-191-5p, miR-223-3p, and miR-338-3p—demonstrates marked upregulation in CIN3, highlighting their potential as sensitive indicators of pre-malignant transformation [236].

Analysis of microarray data has revealed significant miRNA dysregulation in the serum of cervical cancer (CC) patients. Notably, the upregulation of *miR-483-5p*, *miR-1246*, *miR-1275*, and *miR-1290* was initially identified through array screening and subsequently validated by qPCR [237]. This finding is reinforced by independent confirmations of elevated serum levels for several other miRNAs in CC patients compared to healthy controls, including *miR-150*, *miR-221*, *miR-15b*, and the well-characterized *miR-21*. These consistently deregulated circulating miRNAs represent promising candidates for non-invasive diagnostic biomarkers [238,239,240,241].

A defined panel of six urinary miRNAs—comprising *miR-21-5p*, *miR-155-5p*, *miR-199a-5p*, *miR-145-5p*, *miR-218-5p*, and *miR-34a-5p*—has been identified as a promising diagnostic and prognostic tool for cervical pre-cancer and cancer. This signature, which includes molecules with recognized oncogenic and tumor-suppressive functions, demonstrates high sensitivity and specificity in distinguishing disease states. However, further validation through large-scale prospective studies comparing this miRNA panel against conventional cytology and HPV testing is essential before urinary miRNA analysis can be established as a reliable, non-invasive biomarker for cervical cancer screening [242].

Beyond their diagnostic utility, miRNAs demonstrate significant prognostic value in cervical cancer (CC), exhibiting strong correlations with critical clinical parameters including tumor stage, lymph node metastasis, and overall survival. Elevated expression of oncogenic miRNAs, such as *miR-224* and *miR-182*, is associated with advanced disease and unfavorable outcomes, while reduced levels of tumor-suppressive miRNAs, including *miR-150*, *miR-200b*, *miR-636*, *miR-205*, and *miR-187*, correlate with aggressive tumor behavior and diminished survival rates [243]. These associations position miRNA expression profiles as reliable prognostic indicators that can inform clinical decision-making.

The therapeutic potential of miRNAs is supported by pre-clinical evidence demonstrating that modulating miRNA expression—through approaches such as miRNA mimics—can suppress tumor growth, inhibit metastasis, and enhance chemosensitivity in CC models [244]. Bioinformatic and clinical studies further highlight specific miRNAs with dual diagnostic and prognostic significance. For instance, *miR-21* functions as a sensitive diagnostic marker and an oncogenic driver, with upregulation predicting poorer prognosis [245]. Similarly, downregulation of *miR-885-5p* has been linked to disease progression, suggesting its utility as an independent prognostic predictor and therapeutic target [246].

The HPV E4 protein, expressed as an E1-E4 fusion, is predominantly detected in differentiated epithelial cells during the late stage of the viral replication cycle, where it undergoes phosphorylation and cleavage to regulate keratin binding, multimerization, and cytoskeletal disruption. These activities suggest that E4 plays a critical role in viral release and cell cycle regulation, particularly through G2 arrest, thereby facilitating viral propagation while modulating host cellular processes. The PapilloCheck^®^ test and other molecular assays targeting E6 and E7 HPV proteins’ DNA or mRNA offer valuable diagnostic insights, but are limited by high costs and the risk of false-positive results, particularly in transient infections. The assays seem to have the potential to improve screening accuracy and reduce reliance on repeated cytology and invasive procedures [247,248].

#### 3.3.7. Cyclins as Cell Proliferation Biomarkers

Cyclins are key regulators of the cell cycle, controlling transitions between phases by activating cyclin-dependent kinases (CDKs) [249]. The deregulation of cyclin expression, especially cyclin D1, cyclin E, and cyclin A, has been frequently observed in cervical cancer and high-grade cervical intraepithelial neoplasia (CIN2/3) [250] and contributes to uncontrolled cell proliferation.

It was shown that cyclin E and cyclin A overexpression can distinguish high-grade lesions (CIN2+) from low-grade or normal cervical epithelium [251]. Cyclin A and cyclin E overexpression may predict increased recurrence risk after surgical or chemoradiation therapy [252]. High cyclin D1 expression has been correlated with advanced tumor stage, lymph node metastasis, and poorer overall survival in cervical cancer patients. Cyclin expression can also assist in triaging HPV-positive women by identifying those with active cell cycle dysregulation indicative of imminent progression.

The combined assessment of cyclins (e.g., cyclin E and cyclin A) may improve diagnostic accuracy for high-grade lesions. Some studies suggest that cyclin expression profiles, when combined with other molecular markers such as Ki-67 or p16INK4a, provide a stronger prognostic signature than single markers alone [251].

#### 3.3.8. Novel, Promising Biomarkers

##### E2/E6 Ratio

Analysis of the E2/E6 ratio can serve as an indicator of viral integration, distinguishing high-grade from low-grade lesions, though its limited accuracy in advanced disease suggests it is best applied alongside complementary biomarkers such as HPV L1 protein expression [55], with Choi et al.’s 2018 study revealing the association between a decrease in E2/E6 ratio and the lack of HPV L1 expression with CIN2+ lesions [253].

##### LINE-1 ORF1p as Novel Potential Biomarker

ORF1p shows strong potential as a novel biomarker for cervical cancer screening, as it accurately distinguishes dysplastic epithelium from normal cervical epithelium. Its expression was absent or weak in the majority of normal specimens (79.2%), as revealed by Karkas et al. in their 2024 research, and progressively increased with CIN grade, being detectable in 87.5% of CIN1 cases, 63% in CIN 2 cases, 92.8% of CIN 3 cases, and 93.8% of invasive cancers. Its statistically significant capability of differentiating normal tissue from CIN 1 lesions highlights its diagnostic value at the earliest stage in cervical carcinogenesis, complementing or more likely surpassing other markers such as dual staining 9p16/ki-67) [254]. These findings, although in need of further validation, underscore ORF1p as a promising tool for cervical cancer screening algorithms.

##### Circulating HPV DNA—circHPV-DNA

circHPV-DNA, particularly HPV-16, shows a modest but significant association with cervical cancer, although the performance of HPV-18 DNA is weaker. MicroRNAs such as miR-20a, miR-205, and miR-1246 have been linked to cervical cancer, but their expression profiles are inconsistent across studies. Among blood-based biomarkers, HPV-16 E antibodies and circulating HPV DNA exhibit the strongest association with HPV-related cancers, whereas other markers, such as folate, IGF-1, IGFBP-3, and IFN-γ, lack specificity. Epigenetic alterations, especially DNA methylation of host genes such as *CADM1*, *MAL*, and *FAM19A4*, show high diagnostic accuracy for CIN3 and cervical cancer, with assays like GynTect^®^ demonstrating greater specificity than cytology or HPV genotyping. Furthermore, transcriptomic biomarkers, such as AGK protein and mRNA, are significantly upregulated in cervical cancer, suggesting additional potential for prognostic and screening applications [42,55,255,256,257].

#### 3.3.9. AI and Cervical Cancer Screening

The literature reflects the need for structuring all these new markers for cervical HPV-induced lesions to obtain the best possible sensitivity and specificity. Evidence suggests that this will be facilitated by the integration of artificial intelligence (AI). By integrating AI into cervical cancer screening, as many studies now describe, we will achieve substantial improvements in diagnostic accuracy. Machine learning models have already been tested, and the results have been very promising. The models already tested incorporate hrHPV genotyping, cytology, and gynecological examination. Compared with classical triage methods, AI testing software shows a distinct improvement in predictive performance for detecting CIN2+ lesions [258].

The growing availability of large, high-quality datasets of cervical clinical data has created a strong foundation for training and validating AI-based models. Several datasets are public, well-curated, and accessible (e.g., the Cx22 dataset [259], the ISBI Challenge Database for cytology image segmentation [260,261], the SIPaKMeD dataset [262], and the Harlev datasets [263]) for cytology cell classification based on morphology. In contrast, colposcopy image datasets available remain limited. The Intel & MobileODT Cervical Cancer Screening dataset [264] represents the largest open-access resource, although it is derived from mobile-level colposcopy devices. High-magnification colposcopy datasets remain largely unavailable to the public. The IARC Cervical Cancer Image Bank ARC—International Agency for Research on Cancer [265] is a notable database collected by collaborating specialists using standardized formats, but its scale is relatively modest.

##### AI in Cervical Cytology

Accurate classification of cervical cells is essential for effective screening. Manual cytology is limited by variable accuracy and the need for highly trained cytologists, constraints that are especially problematic in low-resource regions [266]. AI has substantially alleviated these limitations [267]. Over the past decades, numerous classification methods have been proposed, many of which rely on segmentation or texture-based feature extraction. Chankong et al. segmented individual cervical cells into nucleus, cytoplasm, and background, extracting morphological features for multi-label classification with an accuracy exceeding 93% [268]. Mariarputham et al. extracted seven sets of texture features, demonstrating a superior performance [269], while Bora et al. developed an integrated classifier using yielding accuracies of 98.11% at the smear level and 99.01% at the cell level [270]. These approaches reduce workload, minimize observer bias, and improve efficiency.

More recent approaches bypass the need for precise segmentation. Zhang et al. pioneered the use of deep learning and transfer learning for cervical cell classification [271], achieving a superior performance in accuracy (98.3%), AUC (0.99), and specificity (98.3%) compared with traditional algorithms. Shi et al. proposed a graph convolution network exploiting relationships across cell images, achieving outstanding accuracy (98.37%), sensitivity (99.80%), and specificity (99.60%) [272].

The digitization of Pap smears into whole-slide images enhances diagnostic efficiency and reduces pathologists’ workload [273]. In cervical cytology, AI leverages these digital slides to transform screening through automated analysis. It enables rapid triage, prioritizes urgent cases, and accurately classifies cell abnormalities to support risk stratification and clinical decision-making [274]. The development of automated cervical cytology systems is progressing rapidly, with platforms such as BestCyte (CellSolutions), CytoProcessor (DATEXIM), and the Genius Digital Diagnostics System (Hologic) representing the forefront of this innovation [275,276]. In cervical histopathology, AI-driven image analysis remains less developed but shows emerging potential to improve diagnostic accuracy and standardize the classification of HPV-related lesions, including koilocytosis, dysplasia, and carcinoma.

##### AI in Colposcopy

Another AI application that piques interest, in addition to prediction models based on molecular biomarkers and relevant clinical aspects, is the ability to analyze and interpret colposcopic images [275] accurately.

In clinical practice, the agreement between colposcopy impressions and histopathology remains suboptimal, contributing to both misdiagnoses and missed diagnoses. AI-assisted colposcopy systems combine high-definition colonoscopy imaging with image-recognition algorithms trained on annotated images to identify suspicious lesions. Given the subjectivity inherent in colonoscopy interpretation, AI can support primary-care providers in low-resource settings by improving the accuracy of lesion identification, grading, and categorization. Although relatively few studies have evaluated AI performance using smartphone-based colposcope images, the available evidence suggests that these systems may perform, or even surpass, trained medical experts [277,278,279]. AI is also increasingly used to support experienced specialists by enhancing diagnostic consistency, identifying the transformation zone (TZ), and guiding biopsy.

Recent research has concentrated on developing deep learning-based classifiers specifically for magnified cervical images captured with specialized equipment, improving agreement with histopathological diagnoses [280,281]. Given the diversity of colposcopy devices and the frequent lack of standardized image annotations, semi-supervised learning methods that infer cervical dysplasia categories from limited but high-quality image sets have emerged as a promising direction in AI-assisted colposcopy [282].

One of the primary goals of cervical cancer screening is to distinguish between normal/CIN1 and CIN2/3+ lesions, as CIN2/3+ typically requires treatment, while CIN1 often regresses spontaneously and may be managed conservatively. Kim et al. developed a data-driven algorithm using color and texture features, achieving a sensitivity of 74% and a specificity of 90% in differentiating CIN3+ from low-grade lesions and normal tissue [283]. Hu et al. conducted a 7-year longitudinal cohort study of 9406 women. They validated a fast R-CNN-based model, achieving an AUC of 0.91 for CIN2+ diagnostic, outperforming both colposcopy evaluation and traditional cytology [284]. Similarly, Cho et al. developed a binary decision model (“Need-To-Biopsy”) to assist clinicians in determining whether a cervical lesion required biopsy. Their best-performing RESNET-152 model achieved an AUC of 0.947, sensitivity of 85.2%, and specificity of 88.2% [285].

##### Multiplex/AI-Driven Panels

AI succeeded in the cytology interpretation process, and this software also demonstrates its value through enhanced screening precision, as validated in large population-based studies [286,287]. Such promising results underscore the need to integrate new factors, such as vaccination status, smoking habits, concurrent infections, vaginal microbiota composition, and duration of HPV positivity, to further improve outcomes for individualized management and therapeutic strategies [275,287]. A novel deep learning model to predict cervical neoplasia with potential for reducing unnecessary conization was developed using both clinical data and colposcopy images in predicting the patients CIN2+ [288].

The future of cervical cancer prevention is being reshaped by a multi-omics approach, which moves beyond single-marker analysis to build a comprehensive molecular portrait of disease risk. By integrating data from genomics (HPV genotyping), epigenomics (host DNA methylation), transcriptomics (E6/E7 mRNA, microRNAs), and proteomics, advanced multiplex panels now capture the complex biological events driving carcinogenesis. The power of this multi-layered data is fully unlocked through artificial intelligence (AI). Machine learning algorithms are trained to discern critical patterns within these multi-omic datasets, transforming them into precise, individualized risk scores. This synergy of comprehensive biomarker panels and intelligent analytics promises to revolutionize cervical cancer screening, enabling unparalleled accuracy in distinguishing transient infections from progressive pre-cancer and guiding personalized clinical management.

Several multiplex panels have emerged as key tools for risk stratification, including those that investigate proteins, immune markers, and other biomarkers. In a study by Berggrund et al., the authors investigated plasma protein biomarkers that could distinguish invasive cervical cancer cases from healthy controls, with the aim of improving screening and earlier disease detection [289]. As a conclusion, the study demonstrated that out of nearly 100 proteins analyzed, 80 showed elevated levels in cervical cancer cases compared with controls. From these, an 11-protein signature (PTX3, ITGB1BP2, AXIN1, STAMPB, SRC, SIRT2, 4E-BP1, PAPPA, HB-EGF, NEMO, and IL-27) achieved high discriminatory power in the discovery cohort, with a sensitivity of ~0.96 and a specificity of 1.0. However, in the replication cohort, performance declined (sensitivity ~0.78, specificity ~0.56), particularly when distinguishing diagnostic samples from those collected prior to diagnosis, underscoring the need for further validation of this protein panel in prospective settings [289].

Similarly, Koshiol et al. evaluated and demonstrated that multiplex immune marker assays can reliably quantify a broad range of cytokines and chemokines in cervical secretions. Preliminary findings suggest associations between specific immune mediators, HPV infection, and CIN2/3, supporting their potential as biomarkers of disease progression. However, larger studies are needed to validate these observations and clarify their clinical relevance [290].

Technological innovation is also advancing detection methods, as shown by Kuntamung et al., who developed a novel nanocomposite immunosensor for the simultaneous detection of protein biomarkers such as p16^INK4a, p53, and Ki-67. This may aid diagnostics if validated further in clinical settings [291].

This data-driven approach extends to cytology and histopathology.

##### Ethical Aspects of AI in Cervical Cancer Screening and Diagnosis

The integration of artificial intelligence (AI) into cervical cancer screening brings important ethical challenges alongside its clinical potential. Key concerns include algorithmic bias and equity, data privacy and security, accountability and human oversight, and the protection of patient autonomy and trust. Ensuring that technological innovation leads to equitable outcomes requires attention to structural determinants of health, inclusive policy development, and ethical standards for data use and service delivery [292,293].

Without explicit equity frameworks, digital tools can inadvertently amplify existing disparities. Many AI models are trained on datasets dominated by high-income, urban, or ethnically homogeneous populations, limiting their accuracy in diverse geographic or socioeconomic settings. This may result in systematic under- or over-diagnosis in underserved communities, where the burden of cervical cancer is already most significant. Similarly, AI-supported smartphone-based self-sampling programs risk excluding women without reliable digital access, reinforcing gaps in screening coverage [294].

Cervical cancer screening involves highly sensitive reproductive health information and images. The use of digital tools and cloud computing raises significant concerns about data privacy and the potential for misuse, especially in regions with weak data protection laws [295].

Regulatory frameworks remain limited or fragmented in many countries, complicating safe deployment. Rigorous technical validation, including internal and external testing across populations and settings, is essential. Guidelines now emphasize transparency, reproducibility, and independent evaluation before clinical adoption [296].

Another aspect is that AI models function as “black boxes,” making it difficult for clinicians to understand the rationale behind their diagnostic outputs. This lack of explain ability hinders clinical trust and complicates accountability in the event of a misdiagnosis [297].

AI is generally viewed as a decision-support tool to enhance, not replace, clinical judgment. Human oversight remains essential to ensure patient safety and quality assurance, particularly as regulatory frameworks for AI in healthcare are still fragmented.

AI-driven panels would benefit from providing more detailed explanations of the specific algorithms applied and the methods used to validate their performance. Furthermore, incorporating a discussion of the ethical dimensions of AI in medical diagnostics—particularly those concerning data privacy and algorithmic bias—would offer a more comprehensive and balanced perspective within the context of cervical cancer.

Machine learning can integrate this multitude of variables—from traditional cytology to novel immune profiles and HPV genotyping—to generate precise risk scores. While artificial intelligence shows significant potential to synthesize these multifaceted variables and generate precise risk scores for personalized management, its clinical implementation faces substantial barriers, including high costs, a lack of algorithmic transparency, and data privacy concerns that must be addressed for effective integration into routine practice.

#### 3.3.10. Clinical Translation Prospects

Introducing new biomarkers in cervical cancer screening and management represents a significant advance in precision oncology. Potential biomarkers for cervical cancer screening exhibit varying degrees of specificity and sensitivity, underscoring their usefulness for identifying high-grade lesions and cancer (Supplementary Table S1) [105,223,298,299,300,301,302,303,304,305,306,307,308,309,310,311,312]. Diagnostic markers such as p16^INK4a and E6/E7 mRNA improve early detection by identifying high-risk lesions with greater accuracy than traditional cytology, a particular advantage in low-resource settings where subjective interpretation can delay diagnosis. p16INK4a/Ki-67 dual staining, the most robustly validated biomarker in cervical pathology, is already included in guidelines in some regions, and is supported by IVD-certified commercial tests. The Ki-67 dual staining test has strong clinical evidence for the triage of HPV-positive women and is used to identify transforming infections. As we mentioned before, the test has superior sensitivity to cytology and comparable specificity, but is prone to subjective interpretation of staining patterns in some cases; therefore, clear guidelines for scoring systems are needed for universal implementation.

E6/E7 mRNA tests are supported by substantial clinical evidence, demonstrating better specificity than HPV DNA for detecting clinically relevant infections. Some guidelines allow their use as an alternative primary screening method, though DNA-based tests remain predominant globally.

Because the test can be used during early infection, this limits its ability to detect all clinically relevant lesions. The test does not benefit from complete guideline integration, reflecting the need for further large-scale longitudinal evidence.

Prognostic biomarkers, including microRNAs and DNA methylation signatures, provide insight into tumor behavior and help tailor clinical decisions. For example, elevated miR-21 expression may indicate the need for closer monitoring or adjuvant treatment, whereas low-risk biomarker profiles can support more conservative management and reduce unnecessary intervention. Even if microRNAs are biologically compelling, their use remains in an early stage of development. Most studies are retrospective or have small sample sizes, which increases the risk of bias. Large, well-designed prospective studies with diverse ethnic populations are needed to confirm the clinical utility and generalizability of miRNA biomarkers. The studies that dominate the literature are small and heterogeneous, limiting generalizability.

Several emerging biomarkers for cervical cancer screening—particularly DNA methylation signatures and microbiome-based markers—are now progressing beyond exploratory studies into early clinical evaluation.

Methylation markers show strong promise, with robust evidence from cross-sectional and prospective cohort studies, but face several obstacles. DNA methylation markers, such as *FAM19A4/miR124-2*, *CADM1/MAL*, and S5-classifier panels, are currently being assessed in registered human studies. In contrast, microbiome-based biomarkers remain largely confined to observational or pilot studies, with limited movement into formal interventional trials.

The use of the cervical (and vaginal) microbiota as a biomarker for cervical cancer is a rapidly evolving field of research. Currently, the use of cervical microbiota is primarily in the pre-clinical and validation-cohort study stages; it is not yet incorporated into clinical guidelines as a standard screening or diagnostic tool.

Assay variability across laboratories and platforms reduces reproducibility and requires standardization. There are limited data on long-term predictive value, especially across diverse populations and self-sampling contexts. On the other hand, implementation barriers, including cost, specialized equipment, and bioinformatics requirements, limit immediate scalability in routine screening.

**Figure 2:**
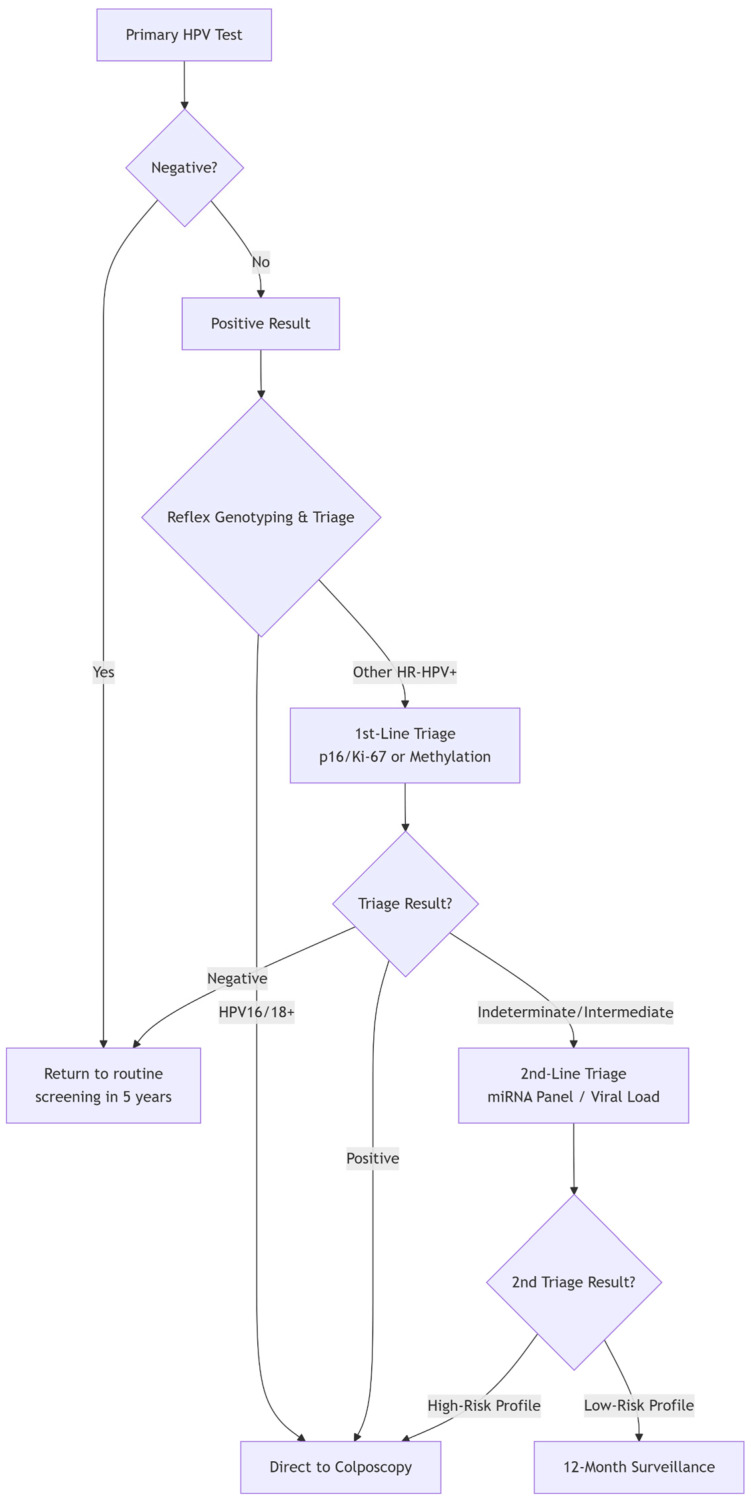
Conceptual integrated clinical workflow combining emerging biomarkers for cervical cancer screening and triage.

A unified pathway is therefore essential to bridge the gap between discovery and practical implementation. By organizing biomarkers by diagnostic purpose, level of evidence, and resource requirements, it is possible to design a layered, stepwise algorithm that maximizes sensitivity for high-risk lesions while minimizing unnecessary procedures. The proposed integrated (Figure 2) workflow outlines how these complementary tools can be sequenced or combined to support screening, triage, and post-treatment surveillance.

This flow chart illustrates a risk-adapted, multi-phase algorithm for cervical cancer screening. Phase 1 begins with a primary HPV Test. Negative results return to routine 5-year screening, while positives undergo reflex genotyping. Women with HPV16/18+ are referred directly to colposcopy. In Phase 2, women with other HR-HPV types receive a 1st-line triage test (e.g., p16/Ki-67 or DNA methylation). A positive result leads to colposcopy; a negative result returns to routine screening. Indeterminate results advance to Phase 3, where a 2nd-line triage (e.g., miRNA panel) resolves risk. A final high-risk profile prompts colposcopy, while a low-risk profile initiates short-term 12-month surveillance.

This pathway efficiently stratifies risk, minimizes unnecessary procedures, and personalizes management through sequential biomarker testing.

Despite the promising trajectory of novel biomarkers, their translation into clinical practice, especially in low-resource settings (LRSs) that bear the highest cervical cancer burden, faces profound practical challenges. The foremost barrier is cost, encompassing not only the price of reagents and kits but also the significant initial investment in specialized equipment (e.g., PCR cyclers, automated stainers, and sequencing platforms), as well as ongoing maintenance and quality-control expenses. This is compounded by substantial infrastructural and technical requirements, including stable electricity, temperature-controlled environments, and a reagent supply chain—all of which are often unreliable in LRS. Furthermore, implementing complex assays such as methylation panels or microbiome analysis requires a skilled technical workforce to perform the tests and interpret the results, creating a human resource gap that is difficult to bridge. Consequently, even highly accurate biomarkers risk becoming inaccessible luxuries, potentially exacerbating global health inequities. For true global impact, future development must prioritize the creation of affordable, point-of-care (POC), or decentralized testing platforms that are robust, minimally instrumented, and require minimal training to operate. Parallel efforts must focus on developing innovative financing models and building local capacity to ensure that technological advances translate into equitable health gains where they are needed most.

The convergence of multi-omics data (genomics, epigenomics, microbiomics) presents a complexity that exceeds traditional analytical methods. Artificial Intelligence (AI), particularly machine learning (ML), offers a powerful toolkit to decipher these high-dimensional datasets and identify subtle, predictive patterns for precise risk stratification.

The application of artificial intelligence (AI) in gynecologic malignancies—including cervical cancer (CCA), endometrial cancer (EC), and ovarian cancer (OC)—has advanced substantially in recent years, offering transformative tools for early screening, diagnosis, therapeutic decision-making, and prognosis prediction. AI technologies are reshaping the conventional paradigms of detection, clinical evaluation, and treatment planning within gynecologic oncology. Despite notable progress, several challenges persist, including variability in data quality, limitations in algorithm interpretability, and barriers to clinical integration. Nevertheless, as technological innovations accelerate and interdisciplinary collaborations deepen, AI is expected to assume an increasingly indispensable role in the precision medicine landscape of gynecologic cancers, ultimately providing patients with more efficient, accurate, and personalized care pathways [313,314].

## 4. Conclusions

Cervical cancer remains a preventable malignancy with significant global disparities in incidence and mortality, largely due to persistent hrHPV infection. While traditional methods such as cytology and histopathology continue to play a key role, their limitations in terms of sensitivity and specificity have spurred the search for more reliable biomarkers. Current evidence supports the clinical application of HPV DNA testing, genotype-specific detection, p16 immunostaining, p16/Ki-67 dual staining, and E6/E7 mRNA assays, all of which improve diagnostic accuracy, reduce unnecessary procedures, and allow better triage of high-risk patients. Prognostic markers such as SCC antigen and telomerase activity further enhance risk stratification, providing valuable insights into tumor aggressiveness, treatment response, and recurrence.

Beyond current standards, a new generation of investigational biomarkers—including DNA methylation panels, microRNAs, and microbiome profiling—holds significant promise for refining risk prediction. Their potential is particularly salient as HPV-vaccinated cohorts enter screening programs, necessitating more precise tools to identify genuine progression risk.

The future of cervical cancer prevention and management lies in integrating validated molecular tools with emerging technologies such as artificial intelligence. This synergic approach will enable personalized management, reduce overtreatment, and ultimately lower the global burden of the disease.

Future research should further investigate the potential of AI-driven approaches in multimodal data integration, real-time dynamic patient monitoring, and broader clinical applicability.

## Figures and Tables

**Table 1:** Biomarker types, detection methods, and clinical uses.

Category	Biomarker/Test	Type/Detection	Clinical Role
A. Guideline-supported/demonstrated efficiency	HPV DNA NAAT (Nucleic Acid Amplification Techniques)	Molecular	Primary screening; high sensitivity for CIN2+; WHO/ASCCP/ACOG endorsed
	hrHPV genotyping	Molecular	Triage; identifies highest-risk infections for immediate colposcopy referral
	Cytology (Pap smear)	Morphology	Still guideline-supported (alone or co-testing with HPV)
	Histopathology (CIN grading; WHO/FIGO classification)	Morphology	Gold standard for diagnosis and staging; CIN1–3 and invasive carcinoma subtyping
	p16INK4a IHC/p16/Ki-67 dual stain (CINtec PLUS)	Immunocytochemistry/ Immunohistochemistry	Triage and diagnostic confirmation; improves specificity for CIN2+; FDA/ASCCP recognition
B. Clinically available/IVD-certified/developing	DNA methylation panels (QIAsure, GynTect, S5 classifier)	Epigenetic/Molecular	Triage of HPV + women; CE-IVD certified; not yet fully in guidelines
	HPV E6/E7 mRNA assays (e.g., Aptima)	Molecular	Triage/adjunct; indicates viral oncogenic activity; guideline-recognized option
	SCC-Ag, Elecsys^®^ SCC (Roche, Basel, Switzerland), ADVIA Centaur SCC, CanAg^®^ SCC EIA (Fujirebio, Tokyo, Japan)	Protein/Serum	Adjunct prognostic marker; mainly for monitoring invasive squamous carcinoma
	Telomerase (hTERT mRNA/protein)	Molecular/Enzyme assay	Investigational, but some clinical labs use it as a diagnostic/prognostic adjunct
	HPV viral load quantification (quantitative PCR, Hybrid Capture 2)	Molecular	Research/adjunct use; heterogeneous data; not standard guideline triage
C. Emerging/research/investigational	Host genetic susceptibility markers (TP53, HLA alleles, cytokine SNPs)	Genetic	Research for risk stratification; may explain why some HPV infections progress
	MCM2/TOP2A (ProExC), HPV E4	Immunohistochemistry/Molecular	Early proliferation markers; investigational adjuncts
	Matrix metalloproteinases (MMPs), TIMPs	Protein/Tumor microenvironment	Prognostic; correlates with progression; research stage
		Cervico-vaginal microbiome profiles (CST classification)	Microbiome sequencing
		MicroRNAs (circulating or cervical)	Non-coding RNA
	Multiplex/AI-driven panels	Multi-omics	Research for predictive algorithms; early validation

**Table 2:** Gene variants associated with cervical cancer risk.

Gene/Locus	Variant(s)	Association with Risk/Effect	References
*TP53* (tumor suppressor)	Codon 72 polymorphism (Pro/Arg; *rs1042522*); also other SNPs (*rs12951053*, *rs1642785*)	One of the most studied. The Pro variant (or heterozygous CG/CC genotypes) is associated with increased risk of CIN3+/persistent HPV infections versus the Arg homozygous genotype. Increased risk ratios ~1.3–1.8 in several studies.	Koshiol J et al. [159].
*NQO1*	SNP609 (*rs1800566*)	The allele C of NQO1 SNP609 found over-transmitted in cervical cancer in Caucasian family-based study. Moreover, associations dependent on HPV type.	Hu X et al. [143].
*TNF-α* (Tumor Necrosis Factor alpha)	Promoter polymorphisms, e.g., −308 G > A, −238 G > A	Some studies/meta-analyses show that TNF-α −308 G > A increases susceptibility; −238 variant may reduce risk. But results are inconsistent across populations.	Wang S et al. [160].
*XRCC1* (DNA repair)	G399A (Arg399Gln; *rs25487*)	Associated with increased risk of cervical cancer in multiple studies (e.g., Saudi population). Suggests variant allele increases risk ~1.4–1.7 fold. May interact with other SNPs (e.g., *TP53*) to modify risk.	Al-Harbi NM et al. [161]
*TGFB1*	T10C (Leu10Pro)	In some populations (e.g., Saudi Arabia), T10C shown to be protective (or different frequencies) in CC versus controls [4].	Al-Harbi NM et al. [161]
*HLA* region/MHC (Human Leukocyte Antigen)	Various HLA-Class I and Class II alleles; specific SNPs in *HLA*, amino acids at certain positions (e.g., positions 13, 71 in *HLA-DRB1;* pos 156 in *HLA-B*); novel susceptibility locus *rs73730372* at 6p21.3; *EXOC1* locus at 4q12; *GSDMB* at 17q12	Strong associations. Both risk and protective alleles identified. HLA haplotypes modify risk of CIN3 and cervical cancer—including effect on HPV persistence. Heritability (common variants) estimated ~36% of liability for cervical neoplasia due to genetic variation.	Leo PJ et al. [162].
Other DNA repair/cell cycle genes	*ATM* G1853A, *CDKN1A* (*p21*) C31A, *HDM2* T309G, *XRCC3* C241T	Some show association in specific populations. For example, in a Saudi cohort, *XRCC1* G399A and *TGFB1* T10C reached significance; other variants had weaker or no association.	Alsbeih G et al. [150].

**Table 3:** lncRNA molecular function and regulatory pathway.

Category	lncRNA	Molecular Function	Regulatory Targets/Pathways
Oncogenic	H19	miRNA sponge (miR-143-3p)	*SIRT1* upregulation
	MALAT1	Epigenetic silencing	*miR-124/GRB2* axis
	HOTAIR	Chromatin remodeling, miRNA sponge	miR-148a/HLA-G, SRF/MKL1/STAT3
	CCAT1	Transcriptional activation, miRNA sponge	Wnt/β-catenin, miR-181a-5p/MMP14/HB-EGF
	XIST	miRNA competition	miR-200a/Fus stabilization
	SNHG14	ceRNA mechanism	miR-206/YWHAZ
	SNHG12	miRNA sequestration	miR-125b/STAT3
	SNHG16	Molecular sponge	miR-216-5p/ZEB1
	SNHG20	miRNA decoy	miR-140-5p/ADAM10/MEK/ERK
Tumor Suppressive	MEG3	Protein interaction	STAT3 ubiquitination/degradation
	GAS5	miRNA sponge	miR-196a/FOXO1, miR-205/PTEN
	STXBP5-AS1	Pathway modulation	PTEN activation
	TUSC8	Tumor suppressor	PTEN pathway
	XLOC_010588	Transcriptional regulation	*c-Myc* inhibition

## Data Availability

No new data were created or analyzed in this study. Data sharing is not applicable to this article.

## Supplementary Materials

The following supporting information can be downloaded at: https://www.mdpi.com/article/10.3390/diagnostics15243231/s1, Table S1: Sensitivity and specificity of biomarkers.

## References

[1] Zhang L., Mosquera I., Lucas E., Rol M., Carvalho A., Basu P., CanScreen5 collaborators (2023). CanScreen5, a global repository for breast, cervical and colorectal cancer screening programs. Nat. Med..

[2] Bray F., Laversanne M., Sung H., Ferlay J., Siegel R., Soerjomataram I., Jemal A. (2024). Global cancer statistics 2022: GLOBOCAN estimates of incidence and mortality worldwide for 36 cancers in 185 countries. CA Cancer. J. Clin..

[3] International Agency for Research on Cancer (IARC) Romania Fact Sheet. Global Cancer Observatory (GLOBOCAN) 2022. https://gco.iarc.who.int/media/globocan/factsheets/populations/642-romania-fact-sheet.pdf.

[4] Aggarwal P. (2014). Cervical cancer: Can it be prevented?. World J. Clin. Oncol..

[5] Ngoma M., Autier P. (2019). Cancer prevention: Cervical cancer. Ecancermedicalscience.

[6] Zhao S., Chen H., Zhao F. (2022). Global guidelines for cervical cancer and precancerous lesions treatment: A systematic review. Zhonghua Yi Xue Za Zhi.

[7] Castle P., Murokora D., Perez C., Alvarez M., Quek S.C., Campbell C. (2017). Treatment of cervical intraepithelial lesions. Int. J. Gynecol. Obstet..

[8] Fashedemi O., Ozoemena O., Peteni S., Haruna A., Shai L., Chen A., Rawson F., Cruickshank M., Grant D., Ola O. (2025). Advances in human papillomavirus detection for cervical cancer screening and diagnosis: Challenges of conventional methods and opportunities for emergent tools. Anal. Methods.

[9] Shanmugam G., Jeyaraj G., Sarkar K. (2025). Molecular mechanisms and diagnostic innovations in HPV-associated head and neck squamous cell carcinomas: Insights into integration, epigenetic modifications, and biomarker applications. Oral. Oncol. Rep..

[10] Taylor L., Law K., Hutchinson A., Dennison R., Usher-Smith J. (2023). Acceptability of risk stratification within population-based cancer screening from the perspective of healthcare professionals: A mixed methods systematic review and recommendations to support implementation. PLoS ONE.

[11] Rosendo-Chalma P., Antonio-Véjar V., Ortiz Tejedor J.G., Ortiz Segarra J., Vega Crespo B., Bigoni-Ordóñez G.D. (2024). The Hallmarks of Cervical Cancer: Molecular Mechanisms Induced by Human Papillomavirus. Biology.

[12] Chan C.K., Aimagambetova G., Ukybassova T., Kongrtay K., Azizan A. (2019). Human Papillomavirus Infection and Cervical Cancer: Epidemiology, Screening, and Vaccination—Review of Current Perspectives. J. Oncol..

[13] Zur Hausen H. (1977). Human papilloma viruses and their possible role in squamous cell carcinomas. Curr. Top. Microbiol. Immunol..

[14] Wang X., Huang X., Zhang Y. (2018). Involvement of Human Papillomaviruses in Cervical Cancer. Front. Microbiol..

[15] Bosch F.X., Lorincz A., Muñoz N., Meijer C.J., Shah K.V. (2002). The causal relation between human papillomavirus and cervical cancer. J. Clin. Pathol..

[16] Okunade K.S. (2020). Human papillomavirus and cervical cancer. J. Obstet. Gynaecol..

[17] Bowden S.J., Doulgeraki T., Bouras E.A., Markozannes G., Athanasiou A., Grout-Smith H., Kechagias K.S., Ellis L.B., Zuber V., Chadeau-Hyam M. (2023). Risk factors for human papillomavirus infection, cervical intraepithelial neoplasia and cervical cancer: An umbrella review and follow-up Mendelian randomisation studies. BMC Med..

[18] Garg P., Krishna M., Subbalakshmi A., Ramisetty S., Mohanty A., Kulkarni P., Horne D., Salgia R., Singhal S. (2024). Emerging biomarkers and molecular targets for precision medicine in cervical cancer. Biochim. Biophys. Acta Rev. Cancer.

[19] Pavelescu L.M.N., Mindru D., Vladareanu R., Curici A. (2025). Molecular Insights into HPV-Driven Cervical Cancer: Oncoproteins, Immune Evasion, and Epigenetic Modifications. Microorganisms.

[20] de Villiers E.M., Fauquet C., Broker T.R., Bernard H.U., zur Hausen H. (2004). Classification of papillomaviruses. Virology.

[21] Terai M., Burk R.D. (2001). Characterization of a novel genital human papillomavirus by overlapping PCR: candHPV86 identified in cervicovaginal cells of a woman with cervical neoplasia. J. Gen. Virol..

[22] Muñoz N., Bosch F.X., de Sanjosé S., Herrero R., Castellsagué X., Shah K.V., Snijders P.J., Meijer C.J., International Agency for Research on Cancer Multicenter Cervical Cancer Study Group (2003). Epidemiologic classification of human papillomavirus types associated with cervical cancer. N. Engl. J. Med..

[23] Arbyn M., Tommasino M., Depuydt C., Dillner J. (2014). Are 20 human papillomavirus types causing cervical cancer?. J. Pathol..

[24] Han F., Guo X., Jiang M., Xia N., Gu Y., Li S. (2024). Structural biology of the human papillomavirus. Structure.

[25] Zheng Z., Baker C. (2006). Papillomavirus genome structure, expression, and post-transcriptional regulation. Front. Biosci..

[26] Fletcher S.B.E., Biswas S. (2025). Structural and functional roles of conserved residues of human papillomavirus (HPV) E2 protein and biological consequences. Virol. J..

[27] Harden M., Munger K. (2017). Human papillomavirus molecular biology. Mutat. Res. Rev. Mutat. Res..

[28] Katzenellenbogen R. (2017). Telomerase Induction in HPV Infection and Oncogenesis. Viruses.

[29] Pańczyszyn A., Boniewska-Bernacka E., Głąb G. (2018). Telomeres and Telomerase During Human Papillomavirus-Induced Carcinogenesis. Mol. Diagn. Ther..

[30] Guerrieri P., Montemaggi P., Brady L.W., Yaeger T.E. (2013). Cervical Intraepithelial Neoplasia (CIN). Encyclopedia of Radiation Oncology.

[31] Ostör A. (1993). Natural history of cervical intraepithelial neoplasia: A critical review. Int. J. Gynecol. Pathol..

[32] Motamedi M., Böhmer G., Neumann H.H., von Wasielewski R. (2015). CIN III lesions and regression: Retrospective analysis of 635 cases. BMC Infect. Dis..

[33] Bruno M., Cassaro N., Bica F., Boemi S. (2021). Progression of CIN1/LSIL HPV Persistent of the Cervix: Actual Progression or CIN3 Coexistence. Infect. Dis. Obstet. Gynecol..

[34] Anton G., Peltecu G., Socolov D., Cornitescu F., Bleotu C., Sgarbura Z., Teleman S., Iliescu D., Botezatu A., Goia C.D. (2011). Type-specific human papillomavirus detection in cervical smears in Romania. APMIS.

[35] Califf R. (2018). Biomarker definitions and their applications. Exp Biol Med.

[36] Mollarasouli F., Bakirhan N.K., Ozkan S.A. (2022). Introduction to biomarkers. The Detection of Biomarkers.

[37] WHO New Recommendations for Screening and Treatment to Prevent Cervical Cancer. https://www.who.int/news/item/06-07-2021-new-recommendations-for-screening-and-treatment-to-prevent-cervical-cancer.

[38] Zhao F.H., Lin M.J., Chen F., Hu S.Y., Zhang R., Belinson J.L., Sellors J.W., Franceschi S., Qiao Y.L., Castle P.E. (2010). Performance of high-risk human papillomavirus DNA testing as a primary screen for cervical cancer: A pooled analysis of individual patient data from 17 population-based studies from China. Lancet Oncol..

[39] Wentzensen N., von Knebel Doeberitz M. (2007). Biomarkers in cervical cancer screening. Dis. Markers.

[40] Yim E.K., Park J.S. (2007). Biomarkers in cervical cancer. Biomark. Insights.

[41] Tornesello M., Buonaguro L.G.P., Buonaguro F. (2013). Viral and cellular biomarkers in the diagnosis of cervical intraepithelial neoplasia and cancer. Biomed. Res. Int..

[42] Onyango C.G., Ogonda L., Guyah B.E.A., Shiluli C., Ganda G., Orang’o O.E., Patel K. (2020). Novel biomarkers with promising benefits for diagnosis of cervical neoplasia: A systematic review. Infect. Agents Cancer.

[43] WHO Human Papillomavirus (HPV) and Cervical Cancer. Fact Sheet, 2020. https://www.who.int/news-room/fact-sheets/detail/human-papilloma-virus-and-cancer.

[44] Cuzick J., Clavel C., Petry K., Meijer C., Hoyer H., Ratnam S., Szarewski A., Birembaut P., Kulasingam S., Sasieni P. (2006). Overview of the European and North American studies on HPV testing in primary cervical cancer screening. Int. J. Cancer.

[45] Clifford G., Smith J., Plummer M.M.N., Franceschi S. (2003). Human papillomavirus types in invasive cervical cancer worldwide: A meta-analysis. Br. J. Cancer.

[46] Zappacosta R., Colasante A., Viola P., D’Antuono T., Lattanzio G., Capanna S., Gatta D.M.P., Rosini S. (2013). Chromogenic in situ hybridization and p16/Ki67 dual staining on formalin-fixed paraffin-embedded cervical specimens: Correlation with HPV-DNA test, E6/E7 mRNA test, and potential clinical applications. Biomed. Res. Int..

[47] Stoler M., Wright T., Ferenczy A.E.A., Ranger-Moore J., Fang Q., Kapadia M., Ridder R. (2018). Routine use of adjunctive p16. immunohistochemistry improves diagnostic agreement of cervical biopsy interpretation. Results from the CERTAIN study. Am. J. Surg. Pathol..

[48] Ebisch R., Rijstenberg L., Soltani G.V.D.H.J., Vedder J., Hermsen M., Bosgraaf R., Massuger L., Meijer C., Heideman D.V.K.F., Melchers W. (2022). Adjunctive use of p16 immunohistochemistry for optimizing management of CIN lesions in a high-risk human papillomavirus-positive population. Acta Obstet. Gynecol. Scand..

[49] Mills A., Paquette C., Castle P.E.A., Stoler M.H. (2015). Risk stratification by p16 immunostaining of CIN1 biopsies. A retrospective study of patients from the quadrivalent HPV vaccine trials. Am. J. Surg. Pathol..

[50] Miralpeix E., Genoves J., Maria S.J.E.A. (2017). Usefulness of p16(INK4a) staining for managing histological high grade squamous intraepithelial cervical lesions. Mod. Pathol..

[51] Cuzick J., Adcock R., Carozzi F., Gillio-Tos A., De Marco L., Del Mistro A., Frayle H., Girlando S., Sani C., Confortini M. (2020). Combined use of cytology, p16 immunostaining and genotyping for triage of women positive for high-risk human papillomavirus at primary screening. Int. J. Cancer.

[52] Paul A., Dutta P., Basu K. (2023). Assessment and clinicopathological correlation of p16 expression in cervical squamous cell carcinoma of Indian population: Diagnostic implications. J. Cancer Res. Ther..

[53] da Mata S., Ferreira J.N.I., Esteves S., Esteves G.L.S., Silva F., Saco A., Cochicho D., Cunha M., Del P.M., Ordi J.F.A. (2021). P16 and HPV Genotype Significance in HPV-Associated Cervical Cancer-A Large Cohort of Two Tertiary Referral Centers. Int. J. Mol. Sci..

[54] Mastutik G., Rahniayu A., Arista A., Murtiastutik D., Kurniasari N., Setyaningrum T., Rahaju A., Sulistyani E. (2021). p16INK4A Expression in Condyloma Acuminata Lesions Associated with High-Risk Human Papillomavirus Infection. Asian Pac. J. Cancer Prev..

[55] Khamseh A., Farhadi A., Jalilvand S., Yarandi F.I.N., Ghorbani S., Saadati H., Shirali E., Jazayeri S., Sarvari J. (2025). Analysis of HPV-16 viral load, integration status, and p16 expression in relation to EBV co-infection and cervical lesion severity. Sci. Rep..

[56] Usta Z., Yilmaz Z., Ersoz S., Mungan S., Cobanoglu U., Guven S. (2024). Comparison of ProExC and p16ink4 a Biological Markers in Lesional Smears With the Immunocytochemical Method and Relationship With Human Papillomavirus in Liquid-based Cervicovaginal Specimens. J. Cytol..

[57] Damgaard R., Jenkins D., de Koning M.N., Quint W., Stoler M., Doorbar J., Kahlert J., Gravitt P., Steiniche T., Petersen L. (2022). Performance of HPV E4 and p16INK4a biomarkers in predicting regression of cervical intraepithelial neoplasia grade 2 (CIN2): Protocol for a historical cohort study. BMJ Open.

[58] Song F., Du H., Xiao A., Wang C., Huang X., Yan P., Liu Z., Qu X., Belinson J., Wu R. (2020). Evaluating the Performance of p16INK4. a Immunocytochemistry in Cervical Cancer Screening. Cancer Manag. Res..

[59] He Y., Shi J., Zhao H., Wang Y., Zhang C., Han S., He Q., Li X., Li S., Wang W. (2023). p16INK4A flow cytometry of exfoliated cervical cells: Its role in quantitative pathology and clinical diagnosis of squamous intraepithelial lesions. Clin. Transl. Med..

[60] Hou J., Du H., Wang C., Song F., Qu X., Wu R. (2024). Performance of P16INK4a immunocytochemical stain in facilitating cytology interpretation of HSIL for HPV-positive women aged 50 and above. Front. Oncol..

[61] Miranda-Falconi P., Flores-Peña G., Jiménez-Trejo M., Torres-Paz Y., Reyes-Hernández D., Estrada-Guzmán J., Hernández-Ramírez E., Torres-Torralba E., Rangel-Ordoñez J., Vejar-Galicia D. (2024). Pioneering molecular screening for cervical precursor lesions and cervical cancer in sera. Front. Oncol..

[62] Yu L., Chen X., Liu X., Fei L., Ma H., Tian T., Wang L., Chen S. (2022). Significance of Triple Detection of p16/ki-67 Dual-Staining, Liquid-Based Cytology and HR HPV Testing in Screening of Cervical Cancer: A Retrospective Study. Front. Oncol..

[63] Walts A., Bose S. (2010). Is the expression pattern of BD ProExC the same as Ki-67? A comparative analysis in cervical biopsies. Appl. Immunohistochem. Mol. Morphol..

[64] Pannone G., Rodolico V., Santoro A.E.A., Muzio L.L., Franco R., Botti G., Aquino G., Pedicillo M.C., Cagiano S., Campisi G. (2012). Evaluation of a combined triple method to detect causative HPV in oral and oropharyngeal squamous cell carcinomas: P16 Immunohisto-chemistry, consensus PCR HPV-DNA, and In Situ Hybridization. Infect. Agent. Cancer.

[65] Zhang Q., Kuhn L., Denny L.A., de Souza M., Taylor S., Wright T.C. (2007). Impact of utilizing p16INK4A immune-histo-chemistry on estimated performance of three cervical cancer screening tests. Int. J. Cancer.

[66] Roelens J., Reuschenbach M., von Knebel Doeberitz M., Wentzensen N., Bergeron C., Arbyn M. (2012). p16INK4a immunocytochemistry versus human papillomavirus testing for triage of women with minor cytologic abnormalities: A systematic review and meta-analysis. Cancer Cytopathol..

[67] Carozzi F., Confortini M., Dalla Palma P., Del Mistro A., Gillio-Tos A., De Marco L., Giorgi-Rossi P., Pontenani G., Rosso S., Sani C. (2008). Use of p16-INK4A overexpression to increase the specificity of human papillomavirus testing: A nested substudy of the NTCC randomised controlled trial. Lancet Oncol..

[68] Carozzi F., Gillio-Tos A., Confortini M., Del Mistro A., Sani C., De Marco L., Girlando S., Rosso S., Naldoni C., Dalla Palma P. (2013). Risk of high-grade cervical intraepithelial neoplasia during follow-up in HPV-positive women according to baseline p16-INK4A results: A prospective analysis of a nested substudy of the NTCC randomised controlled trial. Lancet Oncol..

[69] Reuschenbach M., Seiz M., von Knebel Doeberitz C., Vinokurova S., Duwe A., Ridder R., Sartor H., Kommoss F., Schmidt D., von Knebel Doeberitz M. (2012). Evaluation of cervical cone biopsies for coexpression of p16INK4a and Ki-67 in epithelial cells. Int. J. Cancer.

[70] Bergeron C., Orth G. (2023). La prévention du cancer du col utérin [The prevention of cervical cancer]. Med. Sci..

[71] Srisuttayasathien M., Kantathavorn N., Luasiripanthu T., Petchjorm S., Samrarn J., Ittiamornlert P., Krisorakun W., Vanichtantikul A., Wetcho T., Saeloo S. (2024). Correlation between P16/Ki67 in cervical cytology and diagnosis of cervical intraepithelial neoplasia 2-3 in Thai women infected with high-risk types of human papillomavirus. Taiwan. J. Obstet. Gynecol..

[72] El-Zein M., Gotlieb W., Gilbert L., Hemmings R., Behr M.A., Franco E.L., STAIN-IT Study Group (2021). Dual staining for p16/Ki-67 to detect high-grade cervical lesions: Results from the Screening Triage Ascertaining Intraepithelial Neoplasia by Immunostain Testing study. Int. J. Cancer.

[73] Magkana M., Mentzelopoulou P., Magkana E., Pampanos A., Daskalakis G., Domali E., Rodolakis A., Pappa K. (2021). The p16/ki-67 assay is a safe, effective and rapid approach to triage women with mild cervical lesions. PLoS ONE.

[74] White C.M., Bakhiet S., Bates M., Ruttle C., Pilkington L.J., Keegan H., O’Toole S.A., Sharp L., O’Kelly R., Tewari P. (2020). Exposure to tobacco smoke measured by urinary nicotine metabolites increases risk of p16/Ki-67 co-expression and high-grade cervical neoplasia in HPV-positive women: A two-year prospective study. Cancer Epidemiol..

[75] Gothwal M., Nalwa A., Singh P., Yadav G., Bhati M., Samriya N. (2021). Role of Cervical Cancer Biomarkers p16 and Ki67 in Abnormal Cervical Cytological Smear. J. Obstet. Gynaecol. India.

[76] Luo H., Lian Y., Tao H., Zhao Y., Wang Z., Zhou J., Zhang Z., Jiang S. (2024). Relationship between p16/ki67 immunoscores and PAX1/ZNF582 methylation status in precancerous and cancerous cervical lesions in high-risk HPV-positive women. BMC Cancer.

[77] Benevolo M., Ronco G., Mancuso P., Carozzi F., De Marco L., Allia E., Bisanzi S., Rizzolo R., Gustinucci D., Del Mistro A. (2024). NTCC2 Working Group. Comparison of HPV-positive triage strategies combining extended genotyping with cytology or p16/Ki-67 dual staining in the Italian NTCC2 study. EBioMedicine.

[78] Thrall M.J., McCarthy E., Mito J.K., Rao J., Clinical Practice Committee of the American Society of Cytopathology (2025). Triage options for positive high-risk HPV results from HPV-based cervical cancer screening: A review of the potential alternatives to Papanicolaou test cytology. J. Am. Soc. Cytopathol..

[79] Olivas A., Barroeta J., Lastra R. (2023). Overview of Ancillary Techniques in Cervical Cytology. Acta Cytol..

[80] Secosan C., Pasquini A., Zahoi D., Motoc A., Lungeanu D., Balint O., Ilian A., Balulescu L., Grigoras D., Pirtea L. (2022). Role of Dual-Staining p16/Ki-67 in the Management of Patients under 30 Years with ASC-US/L-SIL. Diagnostics.

[81] Li Y., Fu Y., Cheng B., Xie X., Wang X. (2022). A Comparative Study on the Accuracy and Efficacy Between Dalton and CINtec^®^ PLUS p16/Ki-67 Dual Stain in Triaging HPV-Positive Women. Front. Oncol..

[82] Stoler M., Parvu V., Yanson K., Andrews J., Vaughan L. (2023). Risk stratification of HPV-positive results using extended genotyping and cytology: Data from the baseline phase of the Onclarity trial. Gynecol. Oncol..

[83] Macios A., Nowakowski A. (2022). False Negative Results in Cervical Cancer Screening-Risks, Reasons and Implications for Clinical Practice and Public Health. Diagnostics.

[84] Trzeszcz M., Mazurec M., Jach R., Mazurec K., Kotkowska-Szeps I., Kania M., Wantuchowicz M., Wasowska J., Duczek-Polakiewicz M., Rozmus P. (2023). p16/Ki67 dual stain triage versus cytology in primary human papillomavirus-based cervical cancer screening with limited genotyping. J. Med. Virol..

[85] Harper D.M., Paczos T., Ridder R., Huh W.K. (2025). p16/ki-67 dual stain triage of individuals positive for HPV to detect cervical precancerous lesions. Int. J. Cancer.

[86] Ikenberg H., Bergeron C., Schmidt D., Griesser H., Alameda F., Angeloni C., Bogers J., Dachez R., Denton K., Hariri J. (2013). Screening for cervical cancer precursors with p16/Ki-67 dual-stained cytology: Results of the PALMS study. J. Natl. Cancer Inst..

[87] McMenamin M., McKenna M., McDowell A., Dawson C., McKenna R. (2017). Intra- and inter-observer reproducibility of CINtecR PLUS in ThinPrep cytology preparations. Cytopathol..

[88] Wentzensen N., Fetterman B., Tougawa D., Shiffman M., Castle P.E., Wood S.N., Stiemerling E., Poirtas N., Lorely T., Kinney W. (2014). Interobserver reproducibility and accuracy of p16/Ki67 dual-stain cytology in cervical cancer screening. Cancer Cytopathol..

[89] Allia E., Ronco G., Coccia A., Luparia P., Macrì L., Fiorito C., Maletta F., Deambrogio C., Tunesi S., De Marco L. (2015). Interpretation of p16INK4a/Ki-67 dual immunostaining for the triage of human papillomavirus-positive women by experts and nonexperts in cervical cytology. Cancer Cytopathol..

[90] Benevolo M., Allia E., Gustinucci D., Rollo F., Bulletti S., Cesarini E., Passamonti B., Giovagnoli M.R., Carico E., Carozzi F.M. (2017). Interobserver reproducibility of cytologic p16INK4a /Ki-67 dual immunostaining in human papillomavirus-positive women. Cancer Cytopathol..

[91] Kloboves Prevodnik V., Jerman T., Nolde N., Repše Fokter A., Jezeršek S., Pohar Marinšek Ž., Klopčič U., Hutter Čelik S., Gornik Kramberger K., Primic Žakelj M. (2019). Interobserver variability and accuracy of p16/Ki-67 dual immunocytochemical staining on conventional cervical smears. Diagn. Pathol..

[92] White C., Reynolds S., Murphy K., Keegan H., Naik P., O’Brien R., Pilkington L., Sharkey Ochoa I., Glesson G., Russell N. (2024). Performance of the HPV E6/E7 mRNA Aptima HPV assay combined with partial genotyping compared with the HPV DNA Cobas 4800 HPV test for use in primary screening: Results from the CERVIVA HPV primary screening study in Ireland. Int. J. Cancer.

[93] Wang H., Kim G., Cho H., Kim S., Lee D., Park S., Park K., Lee H. (2015). Diagnostic performance of HPV E6/E7, hTERT, and Ki67. *mRNA RT-qPCR* assays on formalin-fixed paraffin-embedded cervical tissue specimens from women with cervical cancer. Exp. Mol. Pathol..

[94] Derbie A., Mekonnen D., Woldeamanuel Y., Van O.X., Abebe T. (2020). HPV E6/E7 mRNA test for the detection of high grade cervical intraepithelial neoplasia (CIN2+): A systematic review. Infect. Agent. Cancer.

[95] Arbyn M., Simon M., Peeters E., Xu L., Meijer C., Berkhof J., Cuschieri K., Bonde J., Ostrbenk V.A., Zhao F. (2021). 2020 list of human papillomavirus assays suitable for primary cervical cancer screening. Clin. Microbiol. Infect..

[96] Downham L., Jaafar I., Rol M., Nyawira N.V., Valls J., Baena A., Zhang L., Gunter M., Arbyn M., Almonte M. (2024). Accuracy of HPV E6/E7 oncoprotein tests to detect high-grade cervical lesions: A systematic literature review and meta-analysis. Br. J. Cancer.

[97] Singini M., Singh E., Bradshaw D., Ramaliba T., Chen W., Motlhale M., Kamiza A., Babb D.V.C., Muchengeti M., Mathew C. (2023). Usefulness of high-risk HPV early oncoprotein (E6 and E7) serological markers in the detection of cervical cancer: A systematic review and meta-analysis. J. Med. Virol..

[98] Xu F., Ran T., Wei Q., Pan R., Chen S., Luo J. (2024). Diagnostic value of HPV E6/E7 mRNA in screening for cervical intraepithelial neoplasia grade 2 or worse: A systematic review and metaanalysis. Oncol. Lett..

[99] Jin X., Liu F., Zhang Y., Ma Y., Yang L., Wang Y., Liu Y. (2023). Diagnostic value of high-risk HPV E6/E7 mRNA in patients with ASCUS. BMC Womens Health.

[100] Zhang J., Liu G., Yang D., Cui X., Wang C., Wang D., Piao H. (2024). Age-specific performance of human papillomavirus E6/E7 mRNA assay versus cytology for primary cervical cancer screening and triage: Community-based screening in China. Front. Cell Infect. Microbiol..

[101] Liu Y., Jin X., Gong Y., Ma Y., Du B., Yang L., Wang Y., Zhu W. (2023). Do women with high-risk HPV E6/E7 mRNA test positivity and NILM cytology need colposcopy?. Infect. Agent. Cancer.

[102] Gupta S., Warke H., Chaudhari H., Mavani P., Katke R., Kerkar S.M.J. (2022). Human Papillomavirus E6/E7 oncogene transcripts as biomarkers for the early detection of cervical cancer. J. Med. Virol..

[103] Liu J., Yang T., Hu Y., Ye C. (2021). The value of HPV E6/E7 mRNA quantitative analysis in distinguishing high-grade cervical squamous intraepithelial lesions from low-grade cervical squamous intraepithelial lesions. J. Virol. Methods.

[104] Ren C., Zhu Y., Yang L., Zhang X., Liu L., Wang Z., Jiang D. (2019). Prognostic and diagnostic validity of p16/Ki-67, HPV E6/E7 mRNA, and HPV DNA in women with ASCUS: A follow-up study. Virol. J..

[105] Kelly H., Benavente Y., Pavon M., De S.S., Mayaud P., Lorincz A. (2019). Performance of DNA methylation assays for detection of high-grade cervical intraepithelial neoplasia (CIN2+): A systematic review and meta-analysis. Br. J. Cancer.

[106] Chan K., Liu S., Lau L., Ngu S., Chu M., Tse K., Cheung A., Ngan H. (2025). PAX1/SOX1 DNA Methylation Versus Cytology and HPV16/18 Genotyping for the Triage of High-Risk HPV-Positive Women in Cervical Cancer Screening: Retrospective Analysis of Archival Samples. Int. J. Obstet. Gynaecol..

[107] Louvanto K., Verhoef L., Pimenoff V., Eriksson T.L.S., Lagheden C., Gray P.S.D., Sumiec E., Nieminen P., Dillner J., Berkhof J. (2024). Low methylation marker levels among human papillomavirus-vaccinated women with cervical high-grade squamous intraepithelial lesions. Int. J. Cancer.

[108] Salta S., Lobo J.M.B., Henrique R.J.C. (2023). DNA methylation as a triage marker for colposcopy referral in HPV-based cervical cancer screening: A systematic review and meta-analysis. Clin. Epigenetics.

[109] Fackler M., Pleas M., Li Y., Soni A., Xing D., Cope L., Ali S., Van L.Q., Van N.C., Pham H. (2024). Discovery and technical validation of high-performance methylated DNA markers for the detection of cervical lesions at risk of malignant progression in low- and middle-income countries. Clin. Epigenetics.

[110] Vieira-Baptista P., Costa M., Hippe J., Sousa C., Schmitz M., Silva A., Hansel A., Preti M. (2024). Evaluation of Host Gene Methylation as a Triage Test for HPV-Positive Women-A Cohort Study. J. Low. Genit. Tract. Dis..

[111] Ren Y., Qin F., Shen L., Li L., Wu Q., Yi P. (2025). Triage of women with a positive HPV DNA test: Evaluating a DNA methylation panel for detecting cervical intraepithelial neoplasia grade 3 and cervical cancer in cervical cytology samples. BMC Cancer.

[112] Chen S., Dai H., Hu S., Gao T., Chen M., Zhou X., Dai L., Zhao X., Zhao F. (2025). Novel triple-target panels utilizing methylation-sensitive restriction enzyme-based quantitative PCR for detecting advanced cervical precancers and cancers among high-risk HPV-positive women. J. Transl. Med..

[113] Vink F.J., Dick S., Heideman D.A.M., De Strooper L.M.A., Steenbergen R.D.M., Lissenberg-Witte B.I., Floore A., Bonde J.H., Oštrbenk Valenčak A., DNTP Group (2021). Classification of high-grade cervical intraepithelial neoplasia by p16INK4a, Ki-67, HPV E4 and FAM19A4/miR124-2 methylation status demonstrates considerable heterogeneity with potential consequences for management. Int. J. Cancer.

[114] Verhoef L., Bleeker M., Polman N., Steenbergen R., Meijer C., Melchers W., Bekkers R., Molijn A., Quint W.V.K.F., Berkhof J. (2022). Performance of DNA methylation analysis of ASCL1, LHX8, ST6GALNAC5, GHSR, ZIC1 and SST for the triage of HPV-positive women: Results from a Dutch primary HPV-based screening cohort. Int. J. Cancer.

[115] Hernández-López R., Lorincz A.T., Torres-Ibarra L., Reuter C., Scibior-Bentkowska D., Warman R., Nedjai B., Mendiola-Pastrana I., León-Maldonado L., Rivera-Paredez B. (2019). Methylation estimates the risk of precancer in HPV-infected women with discrepant results between cytology and HPV16/18 genotyping. Clin. Epigenetics.

[116] Markovina S., Wang S., Henke L., Luke C., Pak S.C., DeWees T., Pfeifer J.D., Schwarz J.K., Liu W., Chen S. (2018). Serum squamous Cell carcinoma antigen as an early indicator of response during therapy of cervical cancer. Br. J. Cancer.

[117] Tony V., Sathyamurthy A., Ramireddy J., Iswarya S., Gowri S., Thomas A., Peedicayil A., Ram T. (2023). Role of squamous cell carcinoma antigen in prognostication, monitoring of treatment response, and surveillance of locally advanced cervical carcinoma. J. Cancer Res. Ther..

[118] Lozza L., Merola M., Fontanelli R., Stefanon B., Seregni E., Bombardieri E.E.A. (1997). Cancer of the uterine cervix:Clinical value of squamous cell carcinoma antigen (SCC) measurements. Anticancer. Res..

[119] Liu D., Wang W., Zeng Z., Liu X., Zhou Y., Wang C., Li X., Hu K. (2023). Elevated pretreatment squamous cell Carcinoma Antigen indicates unfavorable treatment outcomes in cervical cancer patients receiving definitive radiotherapy. Precis. Radiat. Oncol..

[120] Charakorn C., Thadanipon K., Chaijindaratana S., Rattanasiri S., Numthavaj P., Thakkinstian A. (2018). The association between serum squamous cell carcinoma antigen and recurrence and survival of patients with cervical squamous cell carcinoma: A systematic review and meta-analysis. Gynecol. Oncol..

[121] Fu J., Wang W., Wang Y., Liu C., Wang P. (2019). The role of squamous cell carcinoma antigen (SCC Ag) in outcome prediction after concurrent chemoradiotherapy and treatment decisions for patients with cervical cancer. Radiat Oncol.

[122] Zhang G., Miao L., Wu H., Zhang Y., Fu C. (2021). Pretreatment Squamous Cell Carcinoma Antigen (SCC-Ag) as a Predictive Factor for the Use of Consolidation Chemotherapy in Cervical Cancer Patients After Postoperative Extended-Field Concurrent Chemoradiotherapy. Technol. Cancer Res. Treat..

[123] Lai C., Yen T., Ng K. (2012). Molecular imaging in the management of cervical cancer. J. Formos. Med. Assoc..

[124] Oh J., Bae J. (2018). Optimal cutoff level of serum squamous cell carcinoma antigen to detect recurrent cervical squamous cell carcinoma during post-treatment surveillance. Obstet. Gynecol. Sci..

[125] Sandri M., Salvatici M., Mauro C., Radice D., Lentati P., Massaro M., Boveri S., Zorzino L., Landoni F. (2013). Detection of squamous cell carcinoma antigen with two systems in the follow-up of patients with cervical cancer. Int. J. Biol. Markers.

[126] Shi V., Grover S., Huang Y., Thaker P.H., Kuroki L.M., Powell M.A., Mutch D.G., Contreras J.A., Schwarz J.K., Grigsby P.W. (2024). Accuracy of surveillance serum squamous cell carcinoma antigen for cervical cancer recurrence after definitive chemoradiation. Int. J. Gynecol. Cancer.

[127] Horikawa I., Barrett J.C. (2003). Transcriptional regulation of the telomerase hTERT gene as a target for cellular and viral oncogenic mechanisms. Carcinogenesis.

[128] Wisman G.B., De Jong S., Meersma G.J., Helder M.N., Hollema H., De Vries E.G., Keith W.N., Van der Zee A.G. (2000). Telomerase in (pre)neoplastic cervical disease. Hum. Pathol..

[129] Kyo S., Takakura M., Taira T., Kanaya T., Itoh H., Yutsudo M., Ariga H., Inoue M. (2000). Spl cooperates with c-Myc to activate transcription of the human telomerase reverse transcriptase gene (hTERT). Nucleic Acids Res..

[130] Oh S., Song Y., Yim J., Kim T.K. (1999). The Wilms’ tumor 1 tumor suppressor gene represses transcription of the human telomerase reverse transcriptase gene. J. Biol. Chem..

[131] Oh S., Song Y.H., Yim J., Kim T.K. (2000). Identification of Mad as a repressor of the human telomerase (hTERT) gene. Oncogene.

[132] Grandori C., Cowley S.M., James L.P., Eisenman R.N. (2000). The Myc/Max/Mad network and the transcriptional control of cell behavior. Annu. Rev. Cell Dev. Biol..

[133] Li H., Lee T.H., Avraham H. (2002). A novel tricomplex of BRCA1, Nmi, and c-Myc inhibits c-Myc-induced human telomerase reverse transcriptase gene (hTERT) promoter activity in breast cancer. J. Biol. Chem..

[134] Xu D., Wang Q., Gruber A., Bjorkholm M., Chen Z.G., Zaid A., Selivanova G., Peterson C., Wiman K.G., Pisa P. (2000). Downregulation of telomerase reverse transcriptase mRNA expression by wild type p53 in human tumor cells. Oncogene.

[135] Liu M., Zhang Y., Jian Y., Gu L., Zhang D., Zhou H., Wang Y., Xu Z.X. (2024). The regulations of telomerase reverse transcriptase (TERT) in cancer. Cell Death Dis..

[136] Dogan F., Forsyth N.R. (2021). Telomerase Regulation: A Role for Epigenetics. Cancers.

[137] Liu X., Yuan H., Fu B., Disbrow G.L., Apolinario T., Tomaic V., Kelley M.L., Baker C.C., Huibregtse J., Schlegel R. (2005). The E6AP ubiquitin ligase is required for transactivation of the hTERT promoter by the human papillomavirus E6 oncoprotein. J. Biol. Chem..

[138] Nguyen M.L., Nguyen M.M., Lee D., Griep A.E., Lambert P.F. (2003). The PDZ ligand domain of the human papillomavirus type 16 E6 protein is required for E6’s induction of epithelial hyperplasia in vivo. J. Virol..

[139] Hanahan D. (2022). Hallmarks of Cancer: New Dimensions. Cancer Discov..

[140] Yuan X., Larsson C., Xu D. (2019). Mechanisms underlying the activation of TERT transcription and telomerase activity in human cancer: Old actors and new players. Oncogene.

[141] Ali J., Walter M. (2023). Combining old and new concepts in targeting telomerase for cancer therapy: Transient, immediate, complete and combinatory attack (TICCA). Cancer Cell Int..

[142] Lee S.A., Kim J.W., Roh J.W., Choi J.Y., Lee K.M., Yoo K.Y., Song Y.S., Kang D. (2004). Genetic polymorphisms of GSTM1, p21, p53 and HPV infection with cervical cancer in Korean women. Gynecol. Oncol..

[143] Hu X., Zhang Z., Ma D., Huettner P.C., Massad L.S., Nguyen L., Borecki I., Rader J.S. (2010). TP53, MDM2, NQO1, and susceptibility to cervical cancer. Cancer Epidemiol. Biomarkers Prev..

[144] Oliveira S., Ribeiro J., Sousa H., Pinto D., Baldaque I., Medeiros R. (2012). Genetic polymorphisms and cervical cancer development: ATM G5557A and p53bp1 C1236G. Oncol. Rep..

[145] Ma X.D., Cai G.Q., Zou W., Huang Y.H., Zhang J.R., Wang D.T., Chen B.L. (2013). BRIP1 variations analysis reveals their relative importance as genetic susceptibility factor for cervical cancer. Biochem. Biophys. Res. Commun..

[146] Martínez-Nava G.A., Fernández-Niño J.A., Madrid-Marina V., Torres-Poveda K. (2016). Cervical cancer genetic susceptibility: A systematic review and meta-analyses of recent evidence. PLoS ONE.

[147] Thakur N., Hussain S., Nasare V., Das B.C., Basir S.F., Bharadwaj M. (2012). Association analysis of p16 (CDKN2A) and RB1 polymorphisms with susceptibility to cervical cancer in Indian population. Mol. Biol. Rep..

[148] Juko-Pecirep I., Ivansson E.L., Gyllensten U.B. (2011). Evaluation of Fanconi anaemia genes FANCA, FANCC and FANCL in cervical cancer susceptibility. Gynecol. Oncol..

[149] Kim K., Kang S.B., Chung H.H., Kim J.W., Park N.H., Song Y.S. (2008). XRCC1 Arginine194Tryptophan and GGH-401Cytosine/Thymine polymorphisms are associated with response to platinum-based neoadjuvant chemotherapy in cervical cancer. Gynecol. Oncol..

[150] Alsbeih G., Al-Harbi N., El-Sebaie M., Al-Badawi I. (2013). HPV prevalence and genetic predisposition to cervical cancer in Saudi Arabia. Infect. Agent. Cancer.

[151] Yu K.J., Rader J.S., Borecki I., Zhang Z., Hildesheim A. (2009). CD83 polymorphisms and cervical cancer risk. Gynecol. Oncol..

[152] Hu L., Liu J., Chen X., Zhang Y., Liu L., Zhu J., Chen J., Shen H., Qiang F., Hu Z. (2010). CTLA-4 gene polymorphism +49 A/G contributes to genetic susceptibility to two infection-related cancers-hepatocellular carcinoma and cervical cancer. Hum. Immunol..

[153] Yin J., Wen J., Hang D., Han J., Jiang J., Song C., Liu Y., Liu J., Liu L., Zhu L. (2015). Expression quantitative trait loci for CARD8 contributes to risk of two infection-related cancers—Hepatocellular carcinoma and cervical cancer. PLoS ONE.

[154] Kohaar I., Thakur N., Salhan S., Batra S., Singh V., Sharma A., Sodhani P., Das B.C., Sarkar D.P., Bharadwaj M. (2007). TNF α –308G/A Polymorphism as a Risk Factor for HPV Associated Cervical Cancer in Indian Population. Anal. Cell Pathol..

[155] Liu H., Lyu D., Zhang Y., Sheng L., Tang N. (2017). Association Between the IL-6 rs1800795 Polymorphism and the Risk of Cervical Cancer: A Meta-Analysis of 1210 Cases and 1525 Controls. Technol. Cancer Res. Treat..

[156] Torres-Poveda K., Burguete-García A.I., Bahena-Román M., Méndez-Martínez R., Zurita-Díaz M.A., López-Estrada G., Delgado-Romero K., Peralta-Zaragoza O., Bermúdez-Morales V.H., Cantú D. (2016). Risk allelic load in Th2 and Th3 cytokines genes as biomarker of susceptibility to HPV-16 positive cervical cancer: A case control study. BMC Cancer.

[157] Wang S.S., Gonzalez P., Yu K., Porras C., Li Q., Safaeian M., Rodriguez A.C., Sherman M.E., Bratti C., Schiffman M. (2010). Common genetic variants and risk for HPV persistence and progression to cervical cancer. PLoS ONE.

[158] Chen D., Cui T., Ek W.E., Liu H., Wang H., Gyllensten U. (2015). Analysis of the genetic architecture of susceptibility to cervical cancer indicates that common SNPs explain a large proportion of the heritability. Carcinogenesis.

[159] Koshiol J., Hildesheim A., Gonzalez P., Bratti M.C., Porras C., Schiffman M., Herrero R., Rodriguez A.C., Wacholder S., Yeager M. (2009). Common genetic variation in TP53 and risk of human papillomavirus persistence and progression to CIN3/cancer revisited. Cancer Epidemiol. Biomarkers Prev..

[160] Wang S., Sun H., Jia Y., Tang F., Zhou H., Li X., Zhou J., Huang K., Zhang Q., Hu T. (2015). Association of 42 SNPs with genetic risk for cervical cancer: An extensive meta-analysis. BMC Med. Genet..

[161] Al-Harbi N.M., Bin Judia S.S., Mishra K.N., Shoukri M.M., Alsbeih G.A. (2017). Genetic Predisposition to Cervical Cancer and the Association With XRCC1 and TGFB1 Polymorphisms. Int. J. Gynecol. Cancer.

[162] Leo P., Madeleine M., Wang S., Schwartz S., Newell F.P.U., Hemminki K., Hallmans G., Tiews S., Steinberg W., Rader J. (2017). Defining the genetic susceptibility to cervical neoplasia-A genome-wide association study. PLoS Genet..

[163] Chen D., Gyllensten U. (2015). Lessons and implications from association studies and post-GWAS analyses of cervical cancer. Trends Genet..

[164] Chen D., Juko-Pecirep I., Hammer J., Ivansson E., Enroth S., Gustavsson I., Feuk L., Magnusson P.K.E., McKay J.D., Wilander E. (2013). Genome-wide association study of susceptibility loci for cervical cancer. J. Natl. Cancer Inst..

[165] Takeuchi F., Kukimoto I., Li Z., Li S., Li N., Hu Z., Takahashi A., Inoue S., Yokoi S., Chen J.E.A. (2019). Genome-wide association study of cervical cancer suggests a role for ARRDC3 gene in human papillomavirus infection. Hum. Mol. Genet..

[166] Koel M., Vosa U., Lepamets M., Laivuori H., Lemmela S., Daly M., Palta P., Magi R., Laisk T., Biobank Research Team (2021). GWAS meta-analysis and gene expression data link reproductive tract development, immune response and cellular proliferation/apoptosis with cervical cancer and clarify overlap with other cervical phenotypes. medRxiv.

[167] Olafsdottir T., Stacey S., Sveinbjornsson G., Thorleifsson G., Norland K., Sigurgeirsson B., Thorisdottir K., Kristjansson A., Tryggvadottir L., Sarin K.Y. (2021). Loss-of-Function Variants in the Tumor-Suppressor Gene PTPN14 Confer Increased Cancer Risk. Cancer Res..

[168] Mendonça F., Teles A.M., Nascimento M.D.D.S.B., Santos A.P.A.D., Lopes F.F., Paiva A., Brito H.O., Costa R.G.D. (2022). Human Papillomavirus Modulates Matrix Metalloproteinases During Carcinogenesis: Clinical Significance and Role of Viral Oncoproteins. Vivo.

[169] Fan Y.Z., Zhang J.T., Yang H.C., Yang Y.Q. (2002). Expression of MMP-2,TIMP-2 protein and the ratio of MMP-2/TIMP-2 in gallbladder carcinoma and their significance. World J. Gastroenterol..

[170] Branca M., Ciotti M., Giorgi C., Santini D., Di Bonito L., Costa S., Benedetto A., Bonifacio D., Di Bonito P., Paba P. (2006). Matrix metalloproteinase-2 (MMP-2) and its tissue inhibitor (TIMP-2) are prognostic factors in cervical cancer, related to invasive disease but not to high-risk human papillomavirus (HPV) or virus persistence after treatment of CIN. Anticancer. Res..

[171] Amabebe E., Ogidi H., Anumba D.O. (2022). Matrix metalloproteinase-induced cervical extracellular matrix remodelling in pregnancy and cervical cancer. Reprod. Fertil..

[172] Sheu B.C., Lien H.C., Ho H.N., Lin H.H., Chow S.N., Huang S.C., Hsu S.M. (2003). Increased expression and activation of gelatinolytic matrix metalloproteinases is associated with the progression and recurrence of human cervical cancer. Cancer Res..

[173] Zhu L., Zheng X., Du Y., Xing Y., Xu K., Cui L. (2018). Matrix metalloproteinase-7 may serve as a novel biomarker for cervical cancer. Onco Targets Ther..

[174] Pan M., Tian W., Su M., Zhang S., Hai Y. (2025). Causal Relationship Between Matrix Metalloproteinase and Cervical Lesions: A Two-Sample Mendelian Randomization Analysis. Int. J. Womens Health.

[175] Herbster S., Paladino A., de Freitas S., Boccardo E. (2018). Alterations in the expression and activity of extracellular matrix components in HPV-associated infections and diseases. Clinics.

[176] Dunlop A.L., Mulle J.G., Ferranti E.P., Edwards S., Dunn A.B., Corwin E.J. (2015). Maternal Microbiome and Pregnancy Outcomes That Impact Infant Health: A Review. Adv. Neonatal Care.

[177] Mendling W. (2016). Normal and abnormal vaginal microbiota. J. Lab. Med..

[178] Ottman N., Smith H., de Vos W.M., Belzer C. (2012). The function of our microbiota: Who is out there and what do they do?. Front. Cell. Infect. Microbiol..

[179] Ravel J., Gajer P., Abdo Z., Schneider G., Koenig S.M.S., Karlebach S., Gorle R., Russell J., Tacket C., Brotman R. (2011). Vaginal microbiome of reproductive-age women. Proc. Natl. Acad. Sci. USA.

[180] Chee W.J.Y., Chew S.Y., Than L.T.L. (2020). Vaginal microbiota and the potential of Lactobacillus derivatives in maintaining vaginal health. Microb. Cell Fact..

[181] Kremleva E.A., Sgibnev A.V. (2016). Proinflammatory Cytokines as Regulators of Vaginal Microbiota. Bull. Exp. Biol. Med..

[182] Gao Q., Fan T., Luo S., Zheng J., Zhang L., Cao L., Zhang Z., Li L., Huang Z., Zhang H. (2023). Lactobacillus gasseri LGV03 isolated from the cervico-vagina of HPV-cleared women modulates epithelial innate immune responses and suppresses the growth of HPV-positive human cervical cancer cells. Transl. Oncol..

[183] Mitra A., MacIntyre D.A., Marchesi J.R., Lee Y.S., Bennett P.R., Kyrgiou M. (2016). The vaginal microbiota, human papillomavirus infection and cervical intraepithelial neoplasia: What do we know and where are we going next?. Microbiome.

[184] Wang Y., Thakur R., Shen Q., He Y., Chen C. (2023). Influences of vaginal microbiota on human papillomavirus infection and host immune regulation: What we have learned?. Decod. Infect. Transm..

[185] Bonin C.M., Almeida-Lugo L.Z., dos Santos A.R., Padovani C.T.J., Pina A.F.S., Ferreira A.M.T., Fernandes C.E.d.S., Resende J.C.P., Bovo A.C., Tozetti I.A. (2019). Interleukin-17 expression in the serum and exfoliated cervical cells of patients infected with high-risk oncogenic human papillomavirus. Cytokine.

[186] Sharifian K., Shoja Z., Jalilvand S. (2023). The interplay between human papillomavirus and vaginal microbiota in cervical cancer development. Virol. J..

[187] Brotman R., Shardell M., Gajer P., Tracy J., Zenilman J., Ravel J., Grevitt P.E. (2014). Interplay between the temporal dynamics of the vaginal microbiota and human papillomavirus detection. J. Infect. Dis..

[188] Frąszczak K., Barczyński B., Kondracka A. (2022). Does *Lactobacillus* Exert a Protective Effect on the Development of Cervical and Endometrial Cancer in Women?. Cancers.

[189] Lee J., Lee S., Lee H., Song Y., Lee K., Han M., Sung J., Ko G. (2013). Association of the Vaginal Microbiota with Human. Papillomavirus Infection in a Korean Twin Cohort. PLoS ONE.

[190] Huang R., Liu Z., Sun T., Zhu L. (2024). Cervicovaginal microbiome, high-risk HPV infection and cervical cancer: Mechanisms and therapeutic potential. Microbiol. Res..

[191] Stoian I.L., Botezatu A., Fudulu A., Ilea C.G., Socolov D.G. (2023). Exploring Microbiota Diversity in Cervical Lesion Progression and HPV Infection through 16S rRNA Gene Metagenomic Sequencing. J. Clin. Med..

[192] Curty G., Costa R.L., Siqueira J.D., Meyrelles A.I., Machado E.S., Soares E.A., Soares M.A. (2017). Analysis of the cervical microbiome and potential biomarkers from postpartum HIV-positive women displaying cervical intraepithelial lesions. Sci. Rep..

[193] Fong Amaris W.M., de Assumpção P.P., Valadares L.J., Moreira F.C. (2024). Microbiota changes: The unseen players in cervical cancer progression. Front. Microbiol..

[194] Fobian S., Mei X., Crezee J., Snoek B.C., Steenbergen R.D.M., Hu J., Hagen T.L.M.T., Vermeulen L., Stalpers L.K.A., Oei A.L. (2024). Increased human papillomavirus viral load is correlated to higher severity of cervical disease and poorer clinical outcome: A systematic review. J. Med. Virol..

[195] Zhou Y., Shi X., Liu J., Zhang L. (2023). Correlation between human papillomavirus viral load and cervical lesions classification: A review of current research. Front. Med.

[196] Zhou J., Ma B., Ji J., Liao J., Xu H., Hu H. (2025). HPV16/HPV58 viral load is non-linearly correlated with cervical lesions and can be used as a triage marker. Infect. Agents Cancer.

[197] Lorincz A., Castle P., Sherman M., Scott D., Glass A., Wacholder S., Rush B.B., Gravitt P.E., Schussler J.E., Schiffman M. (2002). Viral load of human papillomavirus and risk of CIN3 or cervical cancer. Lancet.

[198] Liu Y., Xu C., Pan J., Sun C., Zhou H., Meng Y. (2021). Significance of the viral load of high-risk HPV in the diagnosis and prediction of cervical lesions: A retrospective study. BMC Womens Health.

[199] Varesano S., Ciccarese G., Paudice M., Mazzocco K., Gaggero G., Ferrero S., Icardi G., Vellone V.G. (2025). Evaluating HPV Viral Load and Multiple Infections for Enhanced Cervical Cancer Risk-Based Assessment. Life.

[200] Cao M., Wang Y., Wang D., Duan Y., Hong W., Zhang N., Shah W., Wang Y., Chen H. (2020). Increased high-risk human papillomavirus viral load is associated with immunosuppressed microenvironment and predicts a worse long-term survival in cervical cancer patients. Am. J. Clin. Path.

[201] Liu X., Yuan H., Fu B., Disbrow G., Apolinario T., Tomaic V., Kelley M., Baker C., Huibregtse J., Schlegel R. (2005). Evaluation of viral load as a triage strategy with primary high-risk human papillomavirus cervical cancer screening. J. Biol. Chem..

[202] Boucher J., Anku-Bertholet C., Temmar R., Pelmus M. (2009). Evaluation of p16INK4a, minichromosome maintenance protein 2, DNA topoisomerase II, ProExC, andp16INK4a/ProExC in cervical squamous intraepithelial lesions. Hum. Pathol..

[203] Walts A.E., Bose S. (2009). p16, Ki-67, and BD ProExC immunostaining: A practical approach for diagnosis S of cervical intraepithelial neoplasia. Hum. Pathol..

[204] Sahasrabuddhe V.V., Luhn P., Wentzensen N. (2011). Human papillomavirus and cervical cancer: Biomarkers for improved prevention efforts. Future Microbiol..

[205] Jeffus S., Atkins K. (2014). Ancillary Diagnostics in Gynecologic Cytology. Surg. Pathol. Clin..

[206] Zeng T., Guan Y., Li Y.K., Wu Q., Tang X.J., Zeng X., Ling H., Zou J. (2021). The DNA replication regulator MCM6: An emerging cancer biomarker and target. Clin. Chim. Acta.

[207] Dixon E., King L., Nelson R., Simkins S., Knapp S., Brough G., Lenz K., Henderson D., Whitehead C., Hessling J. (2017). Characterization and clinical validation of MCM2 and TOP2A monoclonal antibodies in the BD ProEx™ C assay: An immunoassay which detects aberrant S-phase induction in cervical tissue. J. Immunol. Methods.

[208] Alexander R.P., Fang G., Rozowsky J., Snyder M., Gerstein M.B. (2010). Annotating non-coding regions of the genome. Nat. Rev. Genet..

[209] Hosseini E.S., Meryet-Figuiere M., Sabzalipoor H., Kashani H.H., Nikzad H., Asemi Z. (2017). Dysregulated expression of long noncoding RNAs in gynecologic cancers. Mol. Cancer.

[210] Gupta R., Shah N., Wang K., Kim J., Horlings H., Wong D., Tsai M., Hung T., Argani P., Rinn J. (2010). Long non-coding RNA HOTAIR reprograms chromatin state to promote cancer metastasis. Nature.

[211] Prensner J.R., Chinnaiyan A.M. (2011). The emergence of lncRNAs in cancer biology. Cancer Discov..

[212] Ou L., Wang D., Zhang H., Yu Q., Hua F. (2018). Decreased Expression of miR-138-5p by lncRNA H19 in Cervical Cancer Promotes Tumor Proliferation. Oncol. Res..

[213] Liu S., Song L., Zeng S., Zhang L. (2016). MALAT1-miR-124-RBG2 axis is involved in growth and invasion of HR-HPV-positive cervical cancer cells. Tumour Biol..

[214] He J., Huang B., Zhang K., Liu M., Xu T. (2020). Long non-coding RNA in cervical cancer: From biology to therapeutic opportunity. Biomed. Pharmacother..

[215] Shen H., Wang L., Xiong J., Ren C., Gao C., Ding W., Zhu D., Ma D., Wang H. (2019). Long non-coding RNA CCAT1 promotes cervical cancer cell proliferation and invasion by regulating the miR-181a-5p/MMP14 axis. Cell Cycle.

[216] Zhu H., Zheng T., Yu J., Zhou L., Wang L. (2018). LncRNA XIST accelerates cervical cancer progression via upregulating Fus through competitively binding with miR-200a. Biomed. Pharmacother..

[217] Zhang Z., Wu H., Huang Y., Wei Y. (2025). High SNHG expression may contribute to poor cervical cancer prognosis, based on systematic reviews and meta-analyses. BMC Cancer.

[218] Zhang J., Gao Y. (2019). Long non-coding RNA MEG3 inhibits cervical cancer cell growth by promoting degradation of P-STAT3 protein via ubiquitination. Cancer Cell Int..

[219] Yang W., Hong L., Xu X., Wang Q., Huang J., Jiang L. (2017). LncRNA GAS5 suppresses the tumorigenesis of cervical cancer by downregulating miR-196a and miR-205. Tumour Biol..

[220] Shao S., Wang C., Wang S., Zhang H., Zhang Y. (2019). LncRNA STXBP5-AS1 suppressed cervical cancer progression via targeting miR-96-5p/PTEN axis. Biomed. Pharmacother..

[221] Zhu Y., Liu B., Zhang P., Zhang J., Wang L. (2019). LncRNA TUSC8 inhibits the invasion and migration of cervical cancer cells via miR-641/PTEN axis. Cell Biol. Int..

[222] Liao L.M., Sun X.Y., Liu A.W., Wu J.B., Cheng X.L., Lin J.X., Zheng M., Huang L. (2014). Low expression of long noncoding XLOC_010588 indicates a poor prognosis and promotes proliferation through upregulation of c-Myc in cervical cancer. Gynecol. Oncol..

[223] Zhang X., Mao L., Li L., He Z., Wang N., Song Y. (2019). Long noncoding RNA GIHCG functions as an oncogene and serves as a serum diagnostic biomarker for cervical cancer. J. Cancer.

[224] Wang X., Wang G., Zhang L., Cong J., Hou J., Liu C. (2018). LncRNA PVT1 promotes the growth of HPV positive and negative cervical squamous cell carcinoma by inhibiting TGF-β1. Cancer Cell Int..

[225] Yu C.L., Xu X.L., Yuan F. (2019). LINC00511 is associated with the malignant status and promotes cell proliferation and motility in cervical cancer. Biosci. Rep..

[226] Wang M., Ouyang J., Li H. (2019). CERNA2: A predictor for clinical progression and poor prognosis in cervical carcinoma. J. Cell Biochem..

[227] Zhang Q., Zhang Y., Wang Y. (2019). GHET1 acts as a prognostic indicator and functions as an oncogenic lncRNA in cervical cancer. Biosci. Rep..

[228] Zhang X., Zhao X., Li Y., Zhou Y., Zhang Z. (2019). Long noncoding RNA SOX21-AS1 promotes cervical cancer progression by competitively sponging miR-7/VDAC1. J. Cell. Physiol..

[229] Zhang J., Yao T., Lin Z., Gao Y. (2017). Aberrant Methylation of MEG3 Functions as a Potential Plasma-Based Biomarker for Cervical Cancer. Sci. Rep..

[230] Luo W., Wang M., Liu J., Cui X., Wang H. (2020). Identification of a six lncRNAs signature as novel diagnostic biomarkers for cervical cancer. J. Cell. Physiol..

[231] Banno K., Iida M., Yanokura M., Kisu I., Iwata T., Tominaga E., Tanaka K., Aoki D. (2014). MicroRNA in cervical cancer: OncomiRs and tumor suppressor miRs in diagnosis and treatment. Sci. World J..

[232] Valadi H., Ekström K., Bossios A., Sjöstrand M., Lee J.J., Lötvall J.O. (2007). Exosome-mediated transfer of mRNAs and microRNAs is a novel mechanism of genetic exchange between cells. Nat. Cell Biol..

[233] Wilting S.M., van Boerdonk R.A., Henken F.E., Meijer C.J., Diosdado B., Meijer G.A., le Sage C., Agami R., Snijders P.J., Steenbergen R.D. (2010). Methylation-mediated silencing and tumour suppressive function of hsa-miR-124 in cervical cancer. Mol. Cancer.

[234] Wilting S., Verlaat W., Jaspers A., Makazaji N., Agami R., Meijer C., Snijders P., Steenbergen R. (2013). Methylation-mediated transcriptional repression of microRNAs during cervical carcinogenesis. Epigenetics.

[235] Parvizi M., Vaezi M., Jeddi F., Bakhshandeh M., Eghdam-Zamiri R., Mobaraki-Asl N., Esmati E., Karimi A. (2025). The role and diagnostic value of deregulated miRNAs in cervical cancer. Discov. Oncol..

[236] Wittenborn J., Weikert L., Hangarter B., Stickeler E., Maurer J. (2020). The use of micro RNA in the early detection of cervical intraepithelial neoplasia. Carcinogenesis.

[237] Nagamitsu Y., Nishi H., Sasaki T., Takaesu Y., Terauchi F., Isaka K. (2016). Profiling analysis of circulating microRNA expression in cervical cancer. Mol. Clin. Oncol..

[238] Zhang Z., Wang J., Li J., Wang X., Song W. (2018). MicroRNA-150 promotes cell proliferation, migration, and invasion of cervical cancer through targeting PDCD4. Biomed. Pharmacother..

[239] Lu H., Gu X. (2019). MicroRNA-221 inhibits human papillomavirus 16 E1-E2 mediated DNA replication through activating SOCS1/Type I IFN signaling pathway. Int. J. Clin. Exp. Pathol..

[240] Ocadiz-Delgado R., Lizcano-Meneses S., Trejo-Vazquez J.A., Conde-Perezprina J.C., Garrido-Palmas F., Alvarez-Rios E., García-Villa E., Ruiz G., Illades-Aguiar B., Leyva-Vázquez M.A. (2021). Circulating miR-15b, miR-34a and miR-218 as promising novel early low-invasive biomarkers of cervical carcinogenesis. APMIS.

[241] Ruan F., Wang Y.F., Chai Y. (2020). Diagnostic Values of miR-21, miR-124, and M-CSF in Patients With Early Cervical Cancer. Technol. Cancer Res. Treat..

[242] Aftab M., Poojary S.S., Seshan V., Kumar S., Agarwal P., Tandon S., Zutshi V., Das B.C. (2021). Urine miRNA signature as a potential non-invasive diagnostic and prognostic biomarker in cervical cancer. Sci. Rep..

[243] Sharma P.C., Gupta A. (2020). MicroRNAs: Potential biomarkers for diagnosis and prognosis of different cancers. Transl. Cancer Res..

[244] Di Fiore R., Suleiman S., Drago-Ferrante R., Subbannayya Y., Pentimalli F., Giordano A., Calleja-Agius J. (2022). Cancer Stem Cells and Their Possible Implications in Cervical Cancer: A Short Review. Int. J. Mol. Sci..

[245] Deng Z.M., Chen G.H., Dai F.F., Liu S.Y., Yang D.Y., Bao A.Y., Cheng Y.X. (2022). The clinical value of miRNA-21 in cervical cancer: A comprehensive investigation based on microarray datasets. PLoS ONE.

[246] Chauhan P., Pramodh S., Hussain A., Elsori D., Lakhanpal S., Kumar R., Alsaweed M., Iqbal D., Pandey P., Al O.A. (2024). Understanding the role of miRNAs in cervical cancer pathogenesis and therapeutic responses. Front. Cell Dev. Biol..

[247] Yajid A.I., Zakariah M.A., Mat Zin A.A., Othman N.H. (2017). Potential Role of E4 Protein in Human Papillomavirus Screening: A Review. Asian Pac. J. Cancer Prev..

[248] Przybylski M., Pruski D., Millert-Kalińska S., Krzyżaniak M., de Mezer M., Frydrychowicz M., Jach R., Żurawski J. (2023). Expression of E4 Protein and HPV Major Capsid Protein (L1) as A Novel Combination in Squamous Intraepithelial Lesions. Biomedicines.

[249] Pellarin I., Dall’Acqua A., Favero A., Segatto I., Rossi V., Crestan N., Karimbayli J., Belletti B., Baldassarre G. (2025). Cyclin-dependent protein kinases and cell cycle regulation in biology and disease. Signal Transduct. Target. Ther..

[250] Kim Y.T., Zhao M. (2005). Aberrant cell cycle regulation in cervical carcinoma. Yonsei Med. J..

[251] Erlandsson F., Martinsson-Ahlzén H.S., Wallin K.L., Hellström A.C., Andersson S., Zetterberg A. (2006). Parallel cyclin E and cyclin A expression in neoplastic lesions of the uterine cervix. Br. J. Cancer.

[252] Van de Putte G., Kristensen G.B., Lie A.K., Baekelandt M., Holm R. (2004). Cyclins and proliferation markers in early squamous cervical carcinoma. Gynecol. Oncol..

[253] Choi Y.J., Lee A., Kim T.J., Jin H.T., Seo Y.B., Park J.S., Lee S.J. (2018). E2/E6 ratio and L1 immunoreactivity as biomarkers to determine HPV16-positive high-grade squamous intraepithelial lesions (CIN2 and 3) and cervical squamous cell carcinoma. J. Gynecol. Oncol..

[254] Karkas R., Abdullah K.S.A., Kaizer L., Ürmös Á., Raya M., Tiszlavicz L., Pankotai T., Nagy I., Mátés L., Sükösd F. (2025). LINE-1 ORF1p is a Promising Biomarker in Cervical Intraepithelial Neoplasia Degree Assessment. Int. J. Gynecol. Pathol..

[255] Zou C., Lyu Y., Jiang J., Cao Y., Wang M., Sang C., Zhang R., Li H., Liew C.C., Cheng C. (2020). Use of peripheral blood transcriptomic biomarkers to distinguish high-grade cervical squamous intraepithelial lesions from low-grade lesions. Oncol. Lett..

[256] Balachandra S., Kusin S., Lee R., Blackwell J., Tiro J., Cowell L., Chiang C., Wu S., Varma S., Rivera E. (2021). Blood-based biomarkers of human papillomavirus-associated cancers: A systematic review and meta-analysis. Cancer.

[257] Herbst J., Pantel K., Effenberger K., Wikman H. (2022). Clinical applications and utility of cell-free DNA-based liquid biopsy analyses in cervical cancer and its precursor lesions. Br. J. Cancer.

[258] Dong B., Lu Z., Yang T., Wang J., Zhang Y., Tuo X., Wang J., Lin S., Cai H., Cheng H. (2025). Development, validation, and clinical application of a machine learning model for risk stratification and management of cervical cancer screening based on full-genotyping hrHPV test (SMART-HPV): A modelling study. Lancet Reg. Health West. Pac..

[259] Liu G., Ding Q., Luo H., Sha M., Li X., Ju M. (2022). Cx22: A new publicly available dataset for deep learning-based segmentation of cervical cytology images. Comput. Biol. Med..

[260] Lu Z., Carneiro G., Bradley A.P. (2015). An improved joint optimization of multiple level set functions for the segmentation of overlapping cervical cells. IEEE Trans. Image Process..

[261] Lu Z., Carneiro G., Bradley A.P., Ushizima D., Nosrati M.S., Bianchi A.G., Carneiro C.M., Hamarneh G. (2016). Evaluation of three algorithms for the segmentation of overlapping cervical cells. IEEE J. Biomed. Health Inform..

[262] Plissiti M.E., Dimitrakopoulos P., Sfikas G., Nikou C., Krikoni O., Charchanti A. SIPaKMeD: A new dataset for feature and image-based classification of normal and pathological cervical cells in pap smear images. Proceedings of the 2018 25th IEEE International Conference on Image Processing (ICIP).

[263] Jantzen J., Norup J., Dounias G., Bjerregaard B. (2005). Pap-smear benchmark data for pattern classification. NiSIS.

[264] Payette J., Rachleff J., de Graaf C. (2017). Intel and MobileODT Cervical Cancer Screening Kaggle Competition: Cervix Type Classification Using Deep Learning and Image Classification.

[265] IARC Visual Inspection with Acetic Acid (via) Image Bank. https://screening.iarc.fr/cervicalimagebank.php.

[266] Hussain E., Mahanta L.B., Das C.R., Talukdar R.K. (2020). A Comprehensive Study on the Multi-Class Cervical Cancer Diagnostic Prediction on Pap Smear Images Using a Fusion-Based Decision From Ensemble Deep Convolutional Neural Network. Tissue Cell.

[267] Wu T., Lucas E., Zhao F., Basu P., Qiao Y. (2024). Artificial intelligence strengthens cervical cancer screening—Present and future. Cancer Biol. Med..

[268] Chankong T., Theera-Umpon N., Auephanwiriyakul S. (2014). Automatic cervical cell segmentation and classification in Pap smears. Comput. Methods Programs Biomed..

[269] Mariarputham E.J., Stephen A. (2015). Nominated Texture Based Cervical Cancer Classification. Comput. Math. Methods Med..

[270] Bora K., Chowdhury M., Mahanta L.B., Kundu M.K., Das A.K. (2017). Automated Classification of Pap Smear Images to Detect Cervical Dysplasia. Comput. Methods Programs Biomed..

[271] Zhang L., Lu L., Nogues I., Summers R.M., Liu S., Yao J. (2017). DeepPap: Deep Convolutional Networks for Cervical Cell Classification. IEEE J. BioMed. Health Inform..

[272] Shi J., Wang R., Zheng Y., Jiang Z., Zhang H., Yu L. (2021). Cervical Cell Classification With Graph Convolutional Network. Comput. Methods Programs Biomed..

[273] Niazi M.K.K., Parwani A.V., Gurcan M.N. (2019). Digital pathology and artificial intelligence. Lancet Oncol..

[274] Jiang P., Li X., Shen H., Chen Y., Wang L., Chen H., Feng J., Liu J. (2023). A systematic review of deep learning-based cervical cytology screening: From cell identification to whole slide image analysis. Artif. Intell. Rev..

[275] Dellino M., Cerbone M., d’Amati A., Bochicchio M., Laganà A.S., Etrusco A., Malvasi A., Vitagliano A., Pinto V., Cicinelli E. (2024). Artificial Intelligence in Cervical Cancer Screening: Opportunities and Challenges. AI.

[276] Delga A., Goffin F., Kridelka F., Marée R., Lambert C., Delvenne P. (2014). Evaluation of CellSolutions BestPrep^®^ automated thin-layer liquid-based cytology Papanicolaou slide preparation and BestCyte^®^ cell sorter imaging system. Acta Cytol..

[277] Xue Z., Novetsky A.P., Einstein M.H., Marcus J.Z., Befano B., Guo P., Demarco M., Wentzensen N., Long L.R., Schiffman M. (2020). A demonstration of automated visual evaluation of cervical images taken with a smartphone camera. Int. J. Cancer.

[278] Zhang Y., Zall Y., Nissim R., Zimmermann R. (2022). Evaluation of a new dataset for visual detection of cervical precancerous lesions. Expert. Systs. Appl..

[279] Ito Y., Miyoshi A., Ueda Y., Tanaka Y., Nakae R., Morimoto A., Shiomi M., Enomoto T., Sekine M., Sasagawa T. (2022). An artificial intelligence-assisted diagnostic system improves the accuracy of image diagnosis of uterine cervical lesions. Mol. Clin. Oncol..

[280] Yuan C., Yao Y., Cheng B., Cheng Y., Li Y., Li Y., Liu X., Cheng X., Xie X., Wu J. (2020). The application of deep learning based diagnostic system to cervical squamous intraepithelial lesions recognition in colposcopy images. Sci. Rep..

[281] Chen X., Pu X., Chen Z., Li L., Zhao K.N., Liu H., Zhu H. (2023). Application of EfficientNet-B0 and GRU-based deep learning on classifying the colposcopy diagnosis of precancerous cervical lesions. Cancer Med..

[282] Angara S., Guo P., Xue Z., Antani S. Semi-supervised learning for cervical precancer detection. Proceedings of the 2021 IEEE 34th International Symposium on Computer-Based Medical Systems (CBMS).

[283] Kim E., Huang X., Celebi M.E., Schaefer G. (2013). A Data Driven Approach to Cervigram Image Analysis and Classification. Color Medical Image Analysis. Lecture Notes in Computational Vision and Biomechanics 6.

[284] Hu L., Bell D., Antani S., Xue Z., Yu K., Horning M.P., Gachuhi N., Wilson B., Jaiswal M.S., Befano B. (2019). An Observational Study of Deep Learning and Automated Evaluation of Cervical Images for Cancer Screening. J. Natl. Cancer Inst..

[285] Cho B.J., Choi Y.J., Lee M.J., Kim J.H., Son G.H., Park S.H., Kim H.-B., Joo Y.-J., Cho H.-Y., Kyung M.S. (2020). Classification of Cervical Neoplasms on Colposcopic Photography Using Deep Learning. Sci. Rep..

[286] Yang W., Jin X., Huang L., Jiang S., Xu J., Fu Y., Song Y., Wang X., Wang X., Yang Z. (2024). Clinical evaluation of an artificial intelligence-assisted cytological system among screening strategies for a cervical cancer high-risk population. BMC Cancer.

[287] Hou X., Shen G., Zhou L., Li Y., Wang T., Ma X. (2022). Artificial Intelligence in Cervical Cancer Screening and Diagnosis. Front. Oncol..

[288] Madathil S., Dhouib M., Lelong Q., Bourassine A., Monsonego J. (2025). A multimodal deep learning model for cervical pre-cancers and cancers prediction: Development and internal validation study. Comput. Biol. Med..

[289] Berggrund M., Enroth S., Lundberg M., Assarsson E., Stålberg K., Lindquist D., Hallmans G., Grankvist K., Olovsson M., Gyllensten U. (2019). Identification of Candidate Plasma Protein Biomarkers for Cervical Cancer Using the Multiplex Proximity Extension Assay. Mol. Cell. Proteom..

[290] Koshiol J., Sklavos M., Wentzensen N., Kemp T., Schiffman M., Dunn S., Wang S., Walker J., Safaeian M., Zuna R. (2014). Evaluation of a multiplex panel of immune-related markers in cervical secretions: A methodologic study. Int. J. Cancer.

[291] Kuntamung K., Sangthong P., Jakmunee J., Ounnunkad K. (2024). Simultaneous immunodetection of multiple cervical cancer biomarkers based on a signal-amplifying redox probes/polyethyleneimine-coated gold nanoparticles/2D tungsten disulfide/graphene oxide nanocomposite platform. Bioelectrochemistry.

[292] Gomes M., Provaggi E., Pembe A.B., Olaitan A., Gentry-Maharaj A. (2025). Advancing Cervical Cancer Prevention Equity: Innovations in Self-Sampling and Digital Health Technologies Across Healthcare Settings. Diagnostics.

[293] Alshammari A.H., Ishii H., Hirotsu T., Hatakeyama H., Morishita M., di Luccio E. (2024). Bridging the gap in cervical cancer screening for underserved communities: MCED and the promise of future technologies. Front. Oncol..

[294] Istasy P., Lee W.S., Iansavichene A., Upshur R., Gyawali B., Burkell J., Sadikovic B., Lazo-Langner A., Chin-Yee B. (2022). The Impact of Artificial Intelligence on Health Equity in Oncology: Scoping Review. J. Med. Internet Res..

[295] Galani A., Zikopoulos A., Moustakli E., Potiris A., Paraskevaidi M., Arkoulis I., Machairoudias P., Stavrakaki S.M., Kyrgiou M., Stavros S. (2025). Cervical Cancer Screening in the HPV-Vaccinated and Digital Era: Reassessing Strategies in Light of Artificial Intelligence and Evolving Risk. Cancers.

[296] Pannonhalmi Á., Sipos B., Kurucz R.I., Katona G., Kemény L., Csóka I. (2025). Advancing Regulatory Oversight of Medical Device Trials to Align with Clinical Drug Standards in the European Union. Pharmaceuticals.

[297] Giansanti D., Lastrucci A., Pirrera A., Villani S., Carico E., Giarnieri E. (2025). AI in Cervical Cancer Cytology Diagnostics: A Narrative Review of Cutting-Edge Studies. Bioengineering.

[298] Benevolo M., Vocaturo A., Caraceni D., French D., Rosini S., Zappacosta R., Terrenato I., Ciccocioppo L., Frega A., Giorgi Rossi P. (2011). Sensitivity, specificity, and clinical value of human papillomavirus (HPV) E6/E7 mRNA assay as a triage test for cervical cytology and HPV DNA test. J. Clin. Microbiol..

[299] Chen X., Chen C., Liu L., Dai W., Zhang J., Han C., Zhou S. (2022). Evaluation of p16/Ki-67 dual-stain as triage test for high-risk HPV-positive women: A hospital-based cross-sectional study. Cancer Cytopathol..

[300] Xie Q., Zhu L., Zhang L., Fu S. (2025). Clinical application value of different SCC-Ag reference intervals. Medicine.

[301] Porika M., Tippani R., Mohammad A., Bollam S.R., Panuganti S.D., Abbagani S. (2011). Evaluation of serum human telomerase reverse transcriptase as a novel marker for cervical cancer. Int. J. Biol. Markers.

[302] Zhou Y., Liu J., Chen S., Xin X., Xiao M., Qiang X., Zhang L. (2025). Correlation of different HPV genotype viral loads and cervical lesions: A retrospective analysis of 1585 cases. Cancer Cytopathol..

[303] Yin M., Dai J., Sun R., Wang Y. (2025). Diagnostic performance of PAX1 methylation as a biomarker for cervical lesions: A clinical study and meta-analysis. Ann. Med..

[304] Chen Y., Cui Z., Xiao Z., Hu M., Jiang C., Lin Y., Chen Y. (2016). PAX1 and SOX1 methylation as an initial screening method for cervical cancer: A meta-analysis of individual studies in Asians. Ann. Transl. Med..

[305] Rogeri C.D., Silveira H.C.S., Causin R.L., Villa L.L., Stein M.D., de Carvalho A.C., Arantes L.M.R.B., Scapulatempo-Neto C., Possati-Resende J.C., Antoniazzi M. (2018). Methylation of the hsa-miR-124, SOX1, TERT, and LMX1A genes as biomarkers for precursor lesions in cervical cancer. Gynecol. Oncol..

[306] Yang J.P., Yang X.J., Xiao L., Wang Y. (2016). Long noncoding RNA PVT1 as a novel serum biomarker for detection of cervical cancer. Eur. Rev. Med. Pharmacol. Sci..

[307] Liu M.Y., Li N. (2023). The diagnostic value of lncRNA HOTAIR for cervical carcinoma in vaginal discharge and serum. Medicine.

[308] Wan S., Zhao H. (2020). Analysis of diagnostic and prognostic value of lncRNA MEG3 in cervical cancer. Oncol. Lett..

[309] Cao S., Li H., Li L. (2021). LncRNA SNHG17 Contributes to the Progression of Cervical Cancer by Targeting microRNA-375-3p. Cancer Manag. Res..

[310] Farzanehpour M., Mozhgani S.H., Jalilvand S., Faghihloo E., Akhavan S., Salimi V., Azad T.M. (2019). Serum and tissue miRNAs: Potential biomarkers for the diagnosis of cervical cancer. Virol. J..

[311] Liu S.S., Chan K.K.L., Chu D.K.H., Wei T.N., Lau L.S.K., Ngu S.F., Chu M.M.Y., Tse K.Y., Ip P.P.C., Ng E.K.O. (2018). Oncogenic microRNA signature for early diagnosis of cervical intraepithelial neoplasia and cancer. Mol. Oncol..

[312] Ghosh A., Moirangthem A., Dalui R., Ghosh T., Bandyopadhyay A., Dasgupta A., Banerjee U., Jana N., Basu A. (2014). Expression of matrix metalloproteinase-2 and 9 in cervical intraepithelial neoplasia and cervical carcinoma among different age groups of premenopausal and postmenopausal women. J. Cancer Res. Clin. Oncol..

[313] Wang Q., Zhou X., Wu J., Miao W., Shen B., Shi R. (2025). Research progress of artificial intelligence in the early screening, diagnosis, precise treatment and prognosis prediction of three central gynecological malignancies. Front. Oncol..

[314] Pawar M., Pingale S., Kadam K., Jadhav A., Kaul-Ghanekar R. (2025). Artificial intelligence-driven screening, early diagnosis, and treatment strategies for cervical cancer: An overview. Infect. Agent. Cancer.

