# Supplementary material for: Integrating Biomarkers into Cervical Cancer Screening—Advances in Diagnosis and Risk Prediction: A Narrative Review
Source: Diagnostics (Basel). 2025 Dec 17;15(24):3231. doi: 10.3390/diagnostics15243231 (PMC12731993; doi:10.3390/diagnostics15243231)
Supplement: Supplementary file 1 [file diagnostics-15-03231-s001.zip › diagnostics-3935447-supplementary.pdf]

Table S1: Sensitivity and specificity of biomarkers

Biomarker Class / Specific Marker	Reported Performance	Sample Type	Reference
**hrHPV DNA**	Sensitivity: 97.5% % Specificity: 85.1% % (for CIN3+)	Cervical Scrapes	[298]
**p16/Ki-67 Dual-Stain**	Sensitivity: 89.5 % Specificity: 47.2 % (for CIN3+)	Cervical Scrapes	[299]
**SCC-Ag**	Sensitivity: 52.19 % Specificity: 88.26% (for Cancer)	Tissue	[300]
**Telomerase (hTERT)**	Sensitivity: 80.2% Specificity: 90.4% (for Cancer		[301]
**HPV Viral Load (HPV16)**	Sensitivity: 81.52% Specificity: 64.13% (for CIN2+)	Tissue	[302]
**DNA Methylation (PAX1)**	Sensitivity: 88% Specificity: 94% (for Cancer)	Cervical Scrapes	[303]
	Sensitivity: 73% Specificity: 87% (for Cancer)	Tissue	[304]
**DNA Methylation (SOX1)**	Sensitivity: 71% Specificity: 64% (for Cancer)	Tissue	[304]
**DNA Methylation (PAX1/SOX1)**	Sensitivity: 72% Specificity: 77% (for Cancer)	Tissue	[304]
**DNA Methylation miR-124**	Sensitivity: 86.7% Specificity: 61.3% (for Cancer)		[305]
**DNA Methylation pool (CADM1, FAM19A4, MAL, and miR124-2)**	Sensitivity: 75% Specificity: 68% (for CIN2+)<br>Sensitivity: 74% Specificity: 78% (for CIN3+)	Cervical Scrapes	[105]
**lncRNA GIHCG**	Sensitivity: 88.75% Specificity: 88.5% (for Cancer)	Tissue	[223]
**lncRNA PVT1**	Sensitivity: 98.8% Specificity: 71.6% (for Cancer)	Serum	[306]
**lncRNA HOTAIR**	Sensitivity: 92%<br>Specificity: 98% (for Cancer)<br>Sensitivity: 79%<br>Specificity: 94% (for Cancer)	Tissue,<br>Plasma	[307]
**lncRNA MEG3**	Sensitivity: 82.76% Specificity: 75% (for Cancer)	Serum	[308]
**lncRNA SNHG17**	Sensitivity: 84.7% Specificity: 78.2% (for Cancer)	Serum	[309]
**miR-192**	Sensitivity: 75% Specificity: 94.1% (for Cancer)<br>Sensitivity: 75% Specificity: 94.1% (for Cancer)	Tissue;<br>Serum	[310]
**miR-20a**	Sensitivity: 70.7% Specificity: 79.3% (for Cancer)	Tissue	[311]
**miR-141**	Sensitivity: 82.8% Specificity: 91.7% (for Cancer)	Tissue	[311]
**Combined six miRNA signatures (miR-20a, miR-92a and miR-141; lower: miR-183, miR-210 and miR-944)**	Sensitivity: 91.4% Specificity: 87.6% (for Cancer)	Tissue	[311]
**MMP-9**	Sensitivity: 80% Specificity: 90.0% (for Cancer)	Tissue	[312]
**MMP-2**	Sensitivity: 92% Specificity: 79% (for Cancer)	Tissue	[312]
